# Active reconfiguration of cytoplasmic lipid droplets governs migration of nutrient-limited phytoplankton

**DOI:** 10.1126/sciadv.abn6005

**Published:** 2022-11-04

**Authors:** Anupam Sengupta, Jayabrata Dhar, Francesco Danza, Arkajyoti Ghoshal, Sarah Müller, Narges Kakavand

**Affiliations:** ^1^ Physics of Living Matter, Department of Physics and Materials Science, University of Luxembourg, 162A, Avenue de la Faïencerie, 1511 Luxembourg City, Luxembourg.; ^2^ Swiss Nanoscience lnstitute, University of Basel, 82, Klingelbergslrasse, 4056 Basel, Switzerland.

## Abstract

Nutrient availability, along with light and temperature, drives marine primary production. The ability to migrate vertically, a critical trait of motile phytoplankton, allows species to optimize nutrient uptake, storage, and growth. However, this traditional view discounts the possibility that migration in nutrient-limited waters may be actively modulated by the emergence of energy-storing organelles. Here, we report that bloom-forming raphidophytes harness energy-storing cytoplasmic lipid droplets (LDs) to biomechanically regulate vertical migration in nutrient-limited settings. LDs grow and translocate directionally within the cytoplasm, steering strain-specific shifts in the speed, trajectory, and stability of swimming cells. Nutrient reincorporation restores their swimming traits, mediated by an active reconfiguration of LD size and coordinates. A mathematical model of cell mechanics establishes the mechanistic coupling between intracellular changes and emergent migratory behavior. Amenable to the associated photophysiology, LD-governed behavioral shift highlights an exquisite microbial strategy toward niche expansion and resource optimization in nutrient-limited oceans.

## Teaser

Phytoplankton harness reconfigurable lipid droplets to tune swimming behavior in dynamic nutrient landscapes.

## INTRODUCTION

Oceans, today, are undergoing a major makeover because of the warming temperatures, affecting the concentrations and availability of key nutrients across scales (*1*–*3*). High temperatures drive stratification of the ocean column, exacerbating nutrient limitation (*4*, *5*), affecting primary productivity (*6*). Restructuring of the mixed layer exert critical control on the vertical distribution of phytoplankton (*7*, *8*), and the associated community-scale structure and interactions (*9*–*11*), with far-reaching ramifications on marine ecosystems (*2*, *4*, *5*).

Phytoplankton, key photosynthetic microorganisms occupying the base of nearly all aquatic food webs, acclimatize in the emerging nutrient landscapes by repositioning along the vertical column (*11*–*15*). Nutrients, specifically nitrates and phosphates, form the backbone for basic phytoplankton functions, driving synthesis of proteins and DNA; production of energy-rich adenosine triphosphate (ATP); execution of cellular growth and division; photosynthesis; and diel vertical migrations (*14*, *16*). In the collective context, nitrate and phosphate availability, alongside iron, silica, and other key molecules, is a key determinant of phytoplankton bloom dynamics (*17*, *18*). Limitation in nitrate and phosphate levels affects transcriptional patterns resulting in wide-ranging changes in metabolic pathways (*16*). Under diffusion-limited settings, the specificity of nutrient affinity, species-specific allometries of uptake, and storage properties vary along the ocean column (*2*, *19*–*22*). Altered cell size (*23*), enhanced stoichiometric affinity and plasticity (*24*, *25*), and adapted energy storage capacity (*26*–*28*) are among the critical trait shifts that allow phytoplankton to redistribute along the ocean column.

Among different taxa of phytoplankton, raphidophytes are prolific bloom-forming species (*29*, *30*), owing to their high degree of intraspecific variability that enables them adaptive vertical structuring when environmental conditions change. *Heterosigma akashiwo*, the model phytoplankton studied in this work, is one of the most recognized raphidophytes. They are ecologically relevant, specifically for their role in episodic ichthyotoxic blooms, allelopathic effects, and the strain-specific ability to switch trophic strategies to use inorganic nutrients or derive necessary nutrients by ingesting prokaryotes (*31*, *32*). Strains of *H. akashiwo* exhibit high variability in salinity, temperature, and irradiance tolerance (*33*), nutrient utilization (*29*), and diverse and adaptive swimming strategies (*34*–*36*). Consequently, *H. akashiwo* is a steady member of diverse algal communities globally (*34*, *37*), making them a widely investigated and long-standing model system for studying red tides and diel vertical migrations.

Recent studies have indicated that the response of *H. akashiwo* under diverse environmental stressors is well reflected in a range of phytoplankton taxa, including nonmotile diatoms and motile dinoflagellates (*30*, *36*), possibly due to a high degree of clonal diversity. The ability to migrate vertically, either by buoyancy regulation or by swimming, enhances the molecular flux (relative to pure diffusion) and, thereby, critical access of species to nutrient molecules. Motility confers swimming species a competitive advantage in nutrient-depleted environments (*21*, *34*, *38*). However, migration under nutrient constraints is contingent to the uptake-storage trade-offs, alongside uptake capabilities should there be chanced encounters with ephemeral nutrient patches (*39*, *40*).

Energy-rich starch and lipid bodies maintain phytoplankton fitness under nutrient limitation (*26*–*28*). Persistent limitation transforms heavier, short-lived starch bodies into lighter cytoplasmic lipid droplets (LDs), retaining key metabolic processes including amelioration of physiological stress (*41*–*43*). LDs are formed as nutrient levels dip, supplementing cells with energy-rich units, which are used during nutrient-depleted conditions, or for specific functional requirements, for instance, to alleviate high phospholipid demands during formation of membranes (*42*). Intracellular LDs typically form at the endoplasmic reticulum as miniscule droplets, which then separate due to physical dewetting process, and ultimately merge to form large droplets through one of the two well-recognized mechanisms: droplet coalescence and Ostwald ripening (*44*). Yet, how spatiotemporal coordinates of LDs could affect cellular behavior (for instance, swimming speed and stability) remains unexplored. LDs are energetically expensive; however, phytoplankton swimming under nutrient limitation could offset competitive advantage from larger storage capacity (*45*, *46*) because of enhanced molecular encounters and expansion of potential nutrient pools (*40*). While nutrient limitation has been associated with initiation of phytoplankton migration during blooms (*47*–*49*), contrasting reports suggest suppression of motility under depleted settings (*50*, *51*). Confounding environmental factors such as local pH (*35*) can additionally regulate phytoplankton swimming, alongside biomolecular parameters like variations in the cellular ATP (*52*), which has been reported to shift energy allocations to favor cellular motility. The conundrum that phytoplankton face—in risking reduced fitness because of limited growth vis-à-vis higher, migration-driven encounter rates and access to replete environs—relies on the storage and utilization of energy-rich organelles in light of the motility costs. Yet, biomechanical impact of intracellular LD dynamics on swimming behavior and their emerging interrelations with optimal storage-and-trophic strategy remain unknown.

Here, using *H. akashiwo*—a motile gravitactic alga known for red tides and rapid adaptability (*36*, *53*)—as our model phytoplankton, we report that motile phytoplankton harness energy-rich cytoplasmic LDs to govern migratory behavior under persistent nutrient limitation, enabling vertical redistribution and adapted acquisition strategies. Using two genetically distinct *H. akashiwo* strains, CCMP3107 and CCMP452 (hereafter referred to as strains S-1 and S-2, respectively), we show that LDs prominently nucleate as scattered droplets when concentrations of NO_3_^−^ and PO_4_^3−^ remain below detectable levels, as measured across growth stages (which proxy ecologically relevant nutrient levels). We present the nutrient-driven physiological and behavioral alterations in the two *H. akashiwo* strains in parallel to showcase the distinct strain-specific responses that emerge as LDs grow and translocate within the cells of both strains. Prolonged limitation drives LD growth and merging, accompanied by simultaneous translocation—directionally—within the cytosol. LDs accumulate below the cell nucleus, progressively shifting the ballisticity and stability of the swimming cells. While ballisticity refers to how well phytoplankton migration is correlated and directed (against the gravity direction, since *H. akashiwo* is negatively gravitactic), stability measures how quickly cells reorient back to their stable swimming direction once disturbed out of it (*34*, *36*). Nutrient reincorporation restores the swimming properties because of reversal of the LD translocation, highlighting an active biomechanical control of swimming by the reconfigurable LDs. We quantify the biomechanical impact of the spatiotemporal distribution of LDs on swimming behavior by developing a data-based cell mechanics model that confirms the dependence of swimming traits on the LD size and intracellular position. The agreement between our experimental and in silico behavioral data elucidates that LDs play a key mechanistic role in governing phytoplankton migration under nutrient-limited settings. Together with measurements of cellular stress, photophysiology, and selective switching to mixotrophy (observed only in strain S-1), our results account for strain-specific shifts, providing a mechanistic framework of adaptive migration of nutrient-stressed phytoplankton. Last, using a dissipative energy budget, we rationalize that the emergence of energy-storing units and modulation of swimming behavior are coupled traits that may be crucial for the survival and selection of species under the changing nutrient landscapes of future oceans.

## RESULTS

### Nutrient limitation drives genesis and translocation of intracellular LDs

**Fig. 1.**
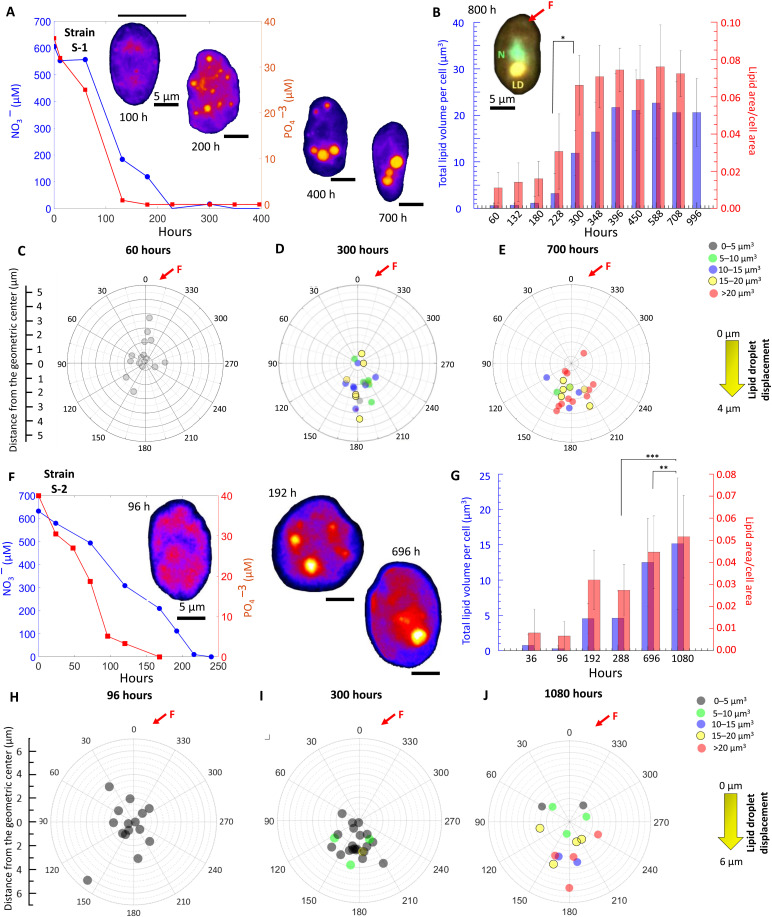
Nutrient depletion drives LD generation and translocation. (**A**) NO_3_^−^ (left axis) and PO_4_^3−^ (right axis) concentrations mediate LD dynamics. Epifluorescent micrograph of Nile Red–stained cells (orange, Materials and Methods): Scattered LDs form large droplets that translocate directionally to position below the nucleus (B, top inset). (**B**) *V*_LD_ (blue, mean ± SD; ANOVA: *P* < 0.001; significant difference at *t* = 228 hours and *t* = 300 hours) and *I*_LD_ (red) increase as stationary stage begins. *I*_LD_ stabilizes at ~0.07, before going further up after 800 hours. Data denote mean ± SD (*t*_60_ = 20, *t*_132_ = 20, *t*_180_ = 21, *t*_228_ = 21, *t*_300_ = 25, *t*_348_ = 30, *t*_396_ = 21, *t*_450_ = 23, *t*_558_ = 24, *t*_708_ = 22). Inset: Multichannel fluorescent micrograph, *t* = 800, shows LD (yellow-orange) located below the nucleus (*N*, light green). (**C** to **E**) Effective LD position relative to *C*_B_: For S-1, *C*_B_ coincides with *N* (center of angular plots), thus with the gravity center, *C*_G_. LD translocates directionally, at *t* = 60 hours (C), 300 hours (D) to 700 hours (E); LD size, angular position, and radial offset from *C*_B_, with the long axis along the 0° to 180° line (fore-aft direction). The propelling flagellum is slightly offset from 0° [red arrow, *F*, (B)], distinguishing fore from the aft direction. (**F**) LD dynamics in S-2 as nutrient levels drop. Inset: LD biogenesis and localization in S-2 at *t* = 96, 192, and 696 hours. (**G**) The S-2 *I*_LD_ (red) reaches a maximum value of 0.052 ± 0.018. Inset: *V*_LD_ (blue) increases at *t* = 288 hours, as S-2 enters stationary stage (*t* test between 696 and 1080 hours; one-way ANOVA between 288, 696, and 1080 hours, *P* < 0.001). (**H** to **J**) Spatiotemporal shift of LDs in individual S-2 cells at *t* = 96 hours (H), 288 hours (I), and 696 hours (J).

We track the growth and translocation of cytoplasmic LDs in *H. akashiwo*, strains S-1 and S-2, using cell-level quantitative imaging, concomitantly measuring the concentrations of NO_3_^−^ and PO_4_^3−^ in the liquid cell cultures (Fig. 1A and Materials and Methods). Depletion of PO_4_^3−^ (*t* ~ 125 hours of inoculation) precedes that of NO_3_^−^ (*t* ~ 225 hours) as the cell populations enter stationary growth stage. LDs, stained with Nile Red and visualized with epifluorescence microscopy (as yellow-orange regions, Materials and Methods), appear initially scattered across the cytosol at *t ~* 200 hours (Fig. 1B), which then enlarge as the NO_3_^−^ availability drops from *C*_0_ ~ 600 μM NO_3_^−^ (~165 pmol/cell; fig. S2A) to *C* ~ 0 μM, 225 hours after cells were inoculated. Similarly, PO_4_^−^ availability drops from *C*_0_ ~ 38 μM (~100 pmol/cell; fig. S2B) to *C* ~ 0 μM in ~135 hours after cells were inoculated. The timing of the LD appearance suggests that it is the sustained limitation of NO_3_^−^, and not PO_4_^3−^, that drives LD growth in our model phytoplankton (*54*). During this same period, the cell area increases, reaching a maximum at the onset of the stationary growth stage (~180 μm^2^), thereafter reducing as populations enter the late stationary stage (*t* > 700 hours; fig. S3). The LD area, normalized by cell area, increases as the NO_3_^−^ availability drops (Fig. 1B), showing a significant enlargement at the onset of stationary stage [inset, Fig. 1B; analysis of variance (ANOVA): *P* < 0.001; asterisk presents statistical difference of lipid volume, *V*_LD_ (per-cell lipid volume)]. During the transition to stationary stage (228 hours < *t* < 300 hours), *V*_LD_ increases threefold, at a rate of 0.12 μm^3^/hour, attaining a steady volume of ~20 μm^3^ into the late stationary stage (area-normalized lipid size, *I*_LD_ ~ 0.07 during this period). Beyond *t* > 800 hours, *V*_LD_ remains stable (Fig. 1B), while *I*_LD_ increases due to the reduction of the cell size (fig. S3). Reduction of cell size, resulting in higher surface-to-volume ratio, suggests that LD accumulation progresses alongside enhancement of diffusion-mediated molecular transport (*23*).

Cells inoculated with lower concentration of nutrients accumulated LDs more rapidly (0.09 μm^3^/hour in 1% *C*_0_ versus 0.076 μm^3^/hour in control condition), although the maximum LD volume generated per cell was lower compared to the control case (*V*_LD_ ~ 10 μm^3^ in 1% *C*_0_ < 22 μm^3^ for controls; figs. S4 and S5). In addition, the carrying capacity of the population (fig. S6) growing in 1% *C*_0_ diminished, demonstrating a strong effect of nutrient concentration on both the growth and lipid formation. Although this is not the focus of the present study, the results indicate that initial nutrient concentrations effectively serve as a proxy for the growth phase of the population. Together, *H. akashiwo* modify growth to adapt the lipid production, yield, and rate dynamically in face of persistent nutrient limitation. The steady volume of energy-rich LDs—despite the nutrient-poor settings during stationary stage (fig. S7)—may equip cells to secure biophysical advantages (*50*, *51*, *55*, *56*) while reserving essential molecules for the maintenance (or modification) of behavioral and physiological traits over longer time scales.

Growing LDs merge to form large droplets (fig. S8), accompanied by translocation of the LDs along the aft-ward direction, resulting in their accumulation below the cell nucleus (*N*; Fig. 1B and Materials and Methods). For S-1, the position of the nucleus, *C*_N_, the heaviest intracellular organelle, coincides with the cell’s center of buoyancy, *C*_B_, thereby overlaying the center of gravity on the *C*_B_ (figs. S9 and S10 and Materials and Methods). Using *C*_B_ as the reference in the wind rose plots presented in Fig. 1 (C to E), we track the radial and angular coordinates of the LDs over time (*C*_L_). During the early exponential stage, LDs are miniscule in size (*V*_LD_ < 5 μm^3^; Fig. 1C) and scattered close to the *C*_B_, shown as short *L*_L_ in fig. S10. However, during the stationary stage, large volume (5 μm^3^ < *V*_LD_ < 20 μm^3^) and their localization below *C*_N_ shift the cell’s center of gravity relative to *C*_B_ due to the lower specific gravity of LDs (~0.9) relative to the surrounding cytosol (~1.05; Fig. 1, C to E, and fig. S10). Furthermore, the larger the LDs, and the offset length, *L*_L_ (distance from the *C*_B_; Table 1), the stronger is rotational moment that affects the cell orientation biomechanically (*36*). During the late stationary stage, LD translocation ceases (Fig. 1, D and E); however, their growing size relative to the cell increases the rotational moments, with biomechanical ramifications on the swimming properties (as will be described later). The aft-ward intracellular mobility of LDs within relatively denser cytosolic environment suggests that the directional translocation of LDs is not a passive gravity-mediated outcome but an active biophysical process.

**Table 1.** Tabulates the mean ± SD (in micrometers) of cell shape, lipid and nucleus shape, and lipid and nucleus position for S-1.

**Growth stage**	**Cell morphology**		**Lipid radius**	**Lipid position**		**Nucleus radius**	**Nucleus position**	
	**Major dia. (*a*)**	**Minor dia. (*b*)**		***X***	***Y***		***X***	***Y***
60 hours	14.81 ± 3.35	9.94 ± 1.02	0.458 ± 0.15	−0.03 ± 0.62	0.81 ± 1.35	1.94 ± 0.18	−0.367 ± 0.24	−0.01 ± 0.355
300 hours	17.4 ± 2.165	10.55 ± 1.34	1.38 ± 0.22	−0.15 ± 0.7	−1.87 ± 1.5	1.97 ± 0.21	0.0002 ± 0.37	−0.39 ± 0.43
700 hours	18.31 ± 1.98	10.78 ± 1.52	1.68 ± 0.18	0.044 ± 1.05	−2.26 ± 1.21	1.88 ± 0.16	−0.06 ± 0.48	0.784 ± 0.41

Measurable differences in the LD growth rate (0.0895 μm^3^/hour for S-1 versus 0.044 μm^3^/hour for S-2), intracellular translocation speeds (0.005 μm/hour for S-1 versus 0.0033 μm/hour for S-2), and cell size modifications were captured in strain S-2 (fig. S3). This intraspecific variability allows strain-specific regulation of swimming properties (Fig. 1, F to J; Table 2; and fig. S11). Strain S-2 shows similar trend as S-1 for the corresponding nutrient depletion (Fig. 1F) and nutrient availability per cell (fig. S2, C and D). While the cell size remains stable, continual growth of LDs increased the lipid volume during the late stationary stage (Fig. 1G). In the LD-free state, S-2 is slightly top heavy (*36*), because *C*_N_ is located above the *C*_B_. As LDs grow and translocate below *C*_N_, the effective center of gravity shifts higher, increasing its offset distance from *C*_B_. This should affect the swimming properties in a manner similar to S-1. However, we observe an intriguing diversion from the expected impact on swimming, thus hinting at an interstrain variability as a survival strategy (discussion on this follows later).

**Table 2.** Tabulates the mean ± SD (in micrometers) of cell shape, lipid, and its position for S-2. Note: Nucleus radii for S-2 cells can be found in Ref (*36*).

**Growth stage**	**Cell morphology**		**Lipid radius**	**Lipid position**	
	**Major dia. (*a*)**	**Minor dia. (*b*)**		***X***	***Y***
96 hours	8.61 ± 0.95	5.02 ± 0.52	0.41 ± 0.29	0.86 ± −1.17	−0.96 ± 1.63
288 hours	8.99 ± 0.92	5.37 ± 0.62	1.03 ± 0.23	−0.32 ± 1.06	−2.25 ± 1.1
696 hours	9.2 ± 1.72	6.13 ± 1.06	1.44 ± 0.2	0.4180 ± 1.66	−2.95 ± 1.57

### Nutrient reincorporation breaks down LDs and reverses cytoplasmic translocation

**Fig. 2.**
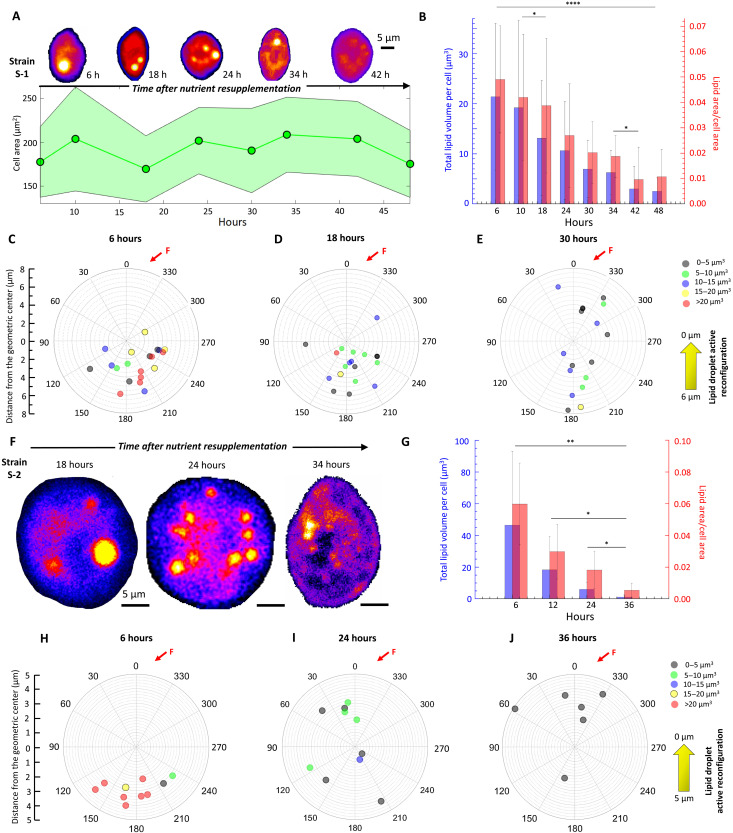
Active reconfiguration of LDs upon nutrient reincorporation. (**A**) Nutrient reincorporation of S-1 leads to LD lipolysis (false color bright spots), while the cell area remains stable over time. LDs translocate from the aft-to-fore direction, reversing the direction of LD mobility under depleted conditions. (**B**) Normalized lipid area, *I*_LD_, reduces from 0.049 ± 0.021 to 0.01 ± 0.01 within ~48 hours (inset shows the total LD volume per cell, *V*_LD_, over time). One-way ANOVA between 10, 18, and 24 hours, *t* test between 34 and 42 hours, *P* < 0.001; asterisks indicate significant difference. (**C** to **E**) Evolution of LD size and coordinates within individual S-1 cells after nutrient reincorporation, shown for *t* = 6 hours (C), 18 hours (D), and 30 hours (E), respectively, measured relative to *C*_B_ (center of the plot). LDs translocate from the bottom to the top of the cell (*F* indicates flagellar position). (**F**) Nutrient reincorporation drives lipolysis and LD translocation along the aft-to-fore direction in S-2. (**G**) *I*_LD_ reduces from 0.06 ± 0.025 before reincorporation to 0.005 ± 0.004 at *t* = 36 hours. Inset shows the total LD volume, *V*_LD_, over time. One-way ANOVA between 12, 24, and 36 hours, *t* test between 24 and 36 hours, *P* < 0.001; asterisks indicate statistically significant difference. (**H** to **J**) LD coordinates relative to *C*_B_ for *t* = 6 hours (H), 24 hours (I), and 36 hours (J) capture the active reconfiguration of the LDs due to nutrient reincorporation.

Nutrient reincorporation reverses LD growth and translocation, accompanied by changes in swimming behavior. Within ~10 hours of reincorporation, LDs shrink and lyse into smaller droplets, which now retrace the limitation-induced LD trajectory to translocate fore-ward within the cell (Fig. 2A). Within the course of the population doubling time, the LDs disappear with no detectable change in cell size (Fig. 2 and figs. S12 and S13), dropping the net lipid volume per cell significantly (Fig. 2B; 10 hours < *t* < 24 hours). The reversal of the LD translocation trajectory (Fig. 2, C to E) shifts back the center of gravity at *C*_B_. The rate of LD lipolysis can be tuned by the concentration of the incorporated nutrients and was found to be more rapid than lipogenesis (fig. S13 and Materials and Methods). Between strains, breakdown of the LDs was faster in S-2, with significant reduction of LDs over 6 to 10 hours of nutrient reincorporation (Fig. 2, F and G). Within ~35 hours, the LDs shrink by 97% of the initial *V*_LD_ (*V*_LD_ < 5 μm^3^) in 80% of the cells chosen randomly (Fig. 2, H to J), eliciting a more uniform lipolytic response compared to the S-1 population. The strain-specific differences, including differential rates of lipolysis and LD retention (fig. S13), combined with the reconfigurability of the LDs—in both size and spatial localization—confirm that cytoplasmic translocation of LDs is an active process driven by the nutrient availability.

### LDs tune phytoplankton motility in a strain-specific manner

**Fig. 3.**
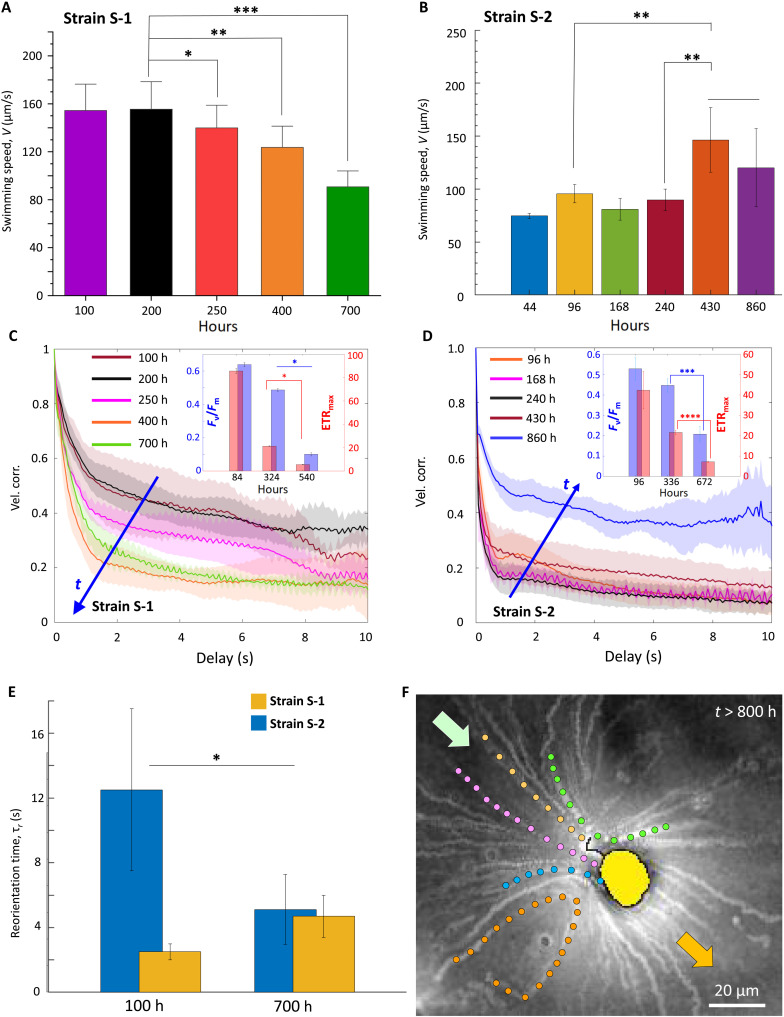
Reconfigurable LDs govern migration of nutrient-limited phytoplankton. (**A**) *V* decreases significantly for nutrient-limited S-1 (*t* > 200 hours). One-way ANOVA, *P* < 0.001, post hoc Tukey’s honest significant difference between exponential (100 and 200 hours), late exponential (250 hours), and stationary (400 and 700 hours) growth stages. Bar plots represent mean *V* ± SD; one asterisk indicates significant difference relative to 100 and 200 hours; two (three) asterisks indicate significance relative to 100, 200, and 250 hours (100, 200, 250, and 400 hours). (**B**) *V* of S-2 (44 to 430 hours) reveals that nutrient-limited population migrates faster against gravity (*t* > 240 hours). One-way ANOVA between 96, 168, 240, and 430 hours, *t* test between 240 and 430 hours, *P* < 0.001; asterisks indicate significant difference. (**C**) Shift from ballistic (*t* = 100 hours) to diffusive (*t* = 700 hours) swimming as populations grow (velocity correlation increases along arrow). Inset: Reduction of *F*_v_/*F*_m_ and ETR_max_; *t* test for *F*_v_/*F*_m_ and ETR_max_, *P* < 0.001 (324 and 540 hours); asterisks indicate statistical significance. (**D**) S-2 velocity correlation increases under nutrient limitation (*t* = 430 hours, MSD plotted in fig. S14). Inset: Drop in *F*_v_/*F*_m_ and the ETR_max_ captured in *t* test (336 and 672 hours, *P* < 0.001); asterisks indicate statistical significance. (**E**) Low τ_r_ indicates high stability at 100 hours (S-1); however, nutrient limitation reduces stability (700 hours). The trend is reversed for S-2. Bar plot represents mean ± SD; asterisk indicates statistical significance. Inset: Joint velocity distribution presents shift in vertical velocity between 100 hours < *t* < 700 hours for S-1; horizontal velocity remains ~0 μm/s. (**F**) S-1 generates feeding current (*t* > 800 hours), visualized as streaks of bacteria and particles getting transported by the current.

Nutrient limitation modifies swimming behavior of motile phytoplankton, amenable to the strain-specific physiological and trophic traits (Fig. 3 and figs. S5, C and D, and S14). We observe a significant alteration in swimming speed and ballisticity for S-1 population inoculated with 1% *C*_0_. For S-1, the swimming speed is progressively arrested as nutrient concentration drops, with the emergence of a low-motility subpopulation (Fig. 3A; figs. S5, C and D, and S16; and Materials and Methods). The corresponding vertical (horizontal) swimming speed ranges around 19.83 ± 24.47 μm/s (30.9 ± 64 μm/s) during the late stationary stage, in contrast to the swimming speed during the exponential stage ranging around 140.34 ± 37 μm/s (62.96 ± 20.8 μm/s). This is shown in figs. S5C (inset) and S16 and discussed in Materials and Methods. At the onset of the stationary stage, the suppression of swimming anisotropy, *I*_A_ (= |*V_y_*/*V_x_*|), from 2.1 to 1.32 synchronizes with significant increment of LD volume (Fig. 1B). Initial nutrient concentration determines how rapidly motility alters (fig. S5C, inset): For concentration *C*_0_ (control), the subpopulation emerges between 200 hours < *t <* 250 hours (Fig. 3A), while for 10% *C*_0_ and 1% *C*_0_ cases, this occurs relatively faster, over 100 hours < *t* < 150 hours (fig. S5C). Consequently, faster drop of *I*_A_ for populations inoculated with 10% and 1% of *C*_0_ (fig. S5C) reveals an inverse dependence of the vertical motility on the LD volume. The behavioral shift from an anisotropic vertical swimming to horizontal swimming (figs. S5D and S16) is in line with migratory adjustments observed under other stressors, including temperature (*34*), light, and turbulence (*36*, *53*). In contrast, S-2 elicits an opposite trend (Fig. 3B and fig. S17) with an enhancement of the swimming speed and the vertical velocity as the population enters the late stationary stage.

The loss of vertical motility is accompanied by a shift from ballistic to diffusive swimming, revealed by the velocity correlations measured over physiological growth stages (Fig. 3C; 100 hours < *t* < 700 hours). Concomitantly, the cells lose orientational stability, confirmed by the increase in the reorientation time, τ_r_, from 2.5 ± 0.5 s (72 hours) to 4.7 ± 1.3 s (700 hours; Fig. 3E, fig. S19, and Materials and Methods). Together with enhanced production of reactive oxygen species (ROS; fig. S18), and the reduced photosynthetic performance (inset, Fig. 3C and Materials and Methods), we infer that the migratory and physiological shifts go hand in hand. Significant drop in the photosynthetic efficiency, *F*_v_/*F*_m_, and the maximum electron transfer rate, ETR_max_, was recorded between 324 hours < *t* < 540 hours (fig. S20, A and C, and Materials and Methods; *t* test, *P* < 0.001; asterisks indicate significant difference), alongside enhanced nonphotochemical quenching (NPQ; fig. S21). Comparing the time scales, we conclude that the photosynthetic machinery is down-regulated under nutrient limitation, following the LD biogenesis and translocation (Fig. 1, A and B), and is correlated with the suppression of vertical swimming and enhanced horizontal migration. Similarly, S-2 down-regulates photosynthetic machinery (fig. S20, B and D), although with a negative correlation with the vertical swimming.

**Fig. 4.**
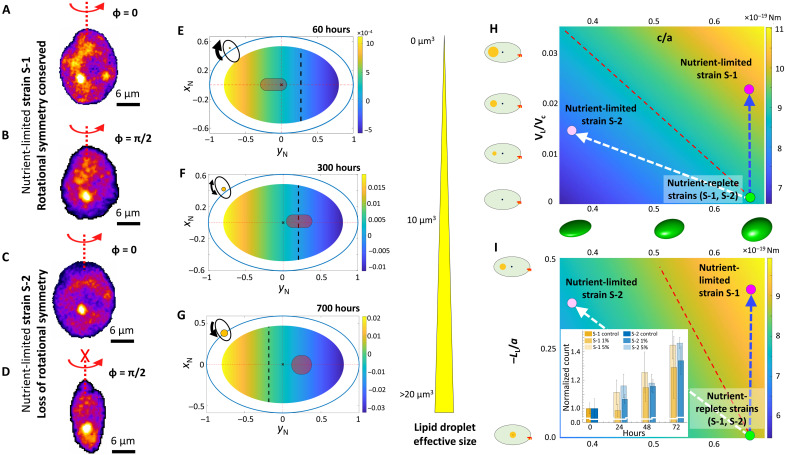
A synergistic interplay of active LD translocation and morphological pliability regulates strain-specific adaptive migration under nutrient limitation. (**A** to **D**) Axisymmetric morphology of S-1 under both nutrient-replete and nutrient-depleted conditions (A and B), whereas S-2 cells turn flat-shaped (C and D), losing rotational symmetry. (**E** to **G**) LD size and position regulates orientational stability of cells (based on Fig. 1, C to E, data), mapped against the reorientation speeds of cells turning from an unstable to a stable orientation. The passive angular velocity (in rad/s) is obtained by varying all possible locations over which LDs reside (changing φ_L_ and *L*_L_, as tabulated in the Supplementary Materials). The angular speed, indicated by color bar, is computed for cytoplasm density (1050 kgm^−3^) (*70*), nucleus density (1300 kgm^−3^) (*71*), and lipid density (900 kgm^−3^) (*72*). The grey patch denotes possible positions where an LD may be observed (plots the mean and the variance from experimental data; Fig. 1, C to E, and fig. S4). The schematic ellipses (top left of the figures) show the effective position of the LDs and the resultant torque (indicated by arrowhead) over the growth phases. (**H** and **I**) The active torque (in Nm) required by a cell to reorient itself as an upward swimmer (negative gravitaxis) for the combination of *c*/*a*, (H) normalized lipid volume, and (I) ratio of *L*_L_ (lipid position from the geometric center) and *a*. The green and magenta circles show nutrient-replete and nutrient-depleted cells, respectively (magenta: S-1, light magenta: S-2). The dashed red line shows the iso-active torque line. Depending on the strain, active torque requirement increases with nutrient limitation, resulting in strain-specific behavioral shifts. Growth following nutrient reincorporation [inset, (I); Materials and Methods] reveals higher growth rate for S-1 [>0.65/day; (*73*)] compared to S-2 [0.4/day; (*36*)] for control case (*C*_0_); however, low concentrations of the initial inoculation (1% *C*_0_ and 5% *C*_0_) suppress the growth rate.

Overall, one can conclude from Figs. 1 and 3 and fig. S20 that it is the formation of LDs that governs the shift in the swimming regime, backed with corresponding changes in the photosynthetic performance. However, as this change is not consistent across the strains, we investigate another important trait, i.e., cell morphology, to arrive at a plausible mechanistic explanation of this apparent inconsistency. In general, we observe from Fig. 3 (as well as Fig. 4) that small LDs do not influence a cell’s orientation stability; however, larger LDs substantially affect cell motility (Materials and Methods).

### Emergent swimming behavior is accompanied by variation of photosynthetic performance

Down-regulation of photophysiology of *H. akashiwo* serves as an energy-conserving adaptive strategy under nutrient constraints (*13*, *53*, *57*); however, it comes at the cost of reduced growth and cell division, necessitating alternative trophic strategies. Prolonged nutrient limitation (*t* > 800 hours) confirms a switch from photo- to phagotrophic mode in S-1, characterized by the loss of motility and generation of feeding currents. The bright streaks, along with a selection of trajectories shown with different hues (Fig. 3F and fig. S22D), visualize bacteria and passive particles trapped within the feeding current. Subsequent encounter and uptake by S-1 enable basic metabolic functions until inorganic nutrients reappear (*13*, *31*, *32*).

Reincorporation of nutrients, even in low concentrations, helps cells to recover the physiological and behavioral traits, bestowing upon cells the crucial ability to execute vertical migration and photosynthesis (fig. S14). The uptake of NO_3_^−^ and PO_4_^3−^ allows nutrient-limited cells for S-1 to ameliorate physiological stress (fig. S23C), recover photophysiology [fig. S23, A and B (top), for S-1 and fig. S23, A and B (bottom), for S-2], and ultimately enhance vertical migration. With just ~^1^/_8_ *C*_0_, S-1 showed up to 28% recovery in the photosynthetic efficiency (fig. S23, A and B, top), along with partial recovery of ballisticity and stability, while S-2 showed recovery even until 48 hours after supplementation [fig. S23, A and B (bottom)]. The concerted restoration of photophysiology and motility, accompanied by reduction of ROS levels (fig. S23), suggests that the cell population is now primed to execute vertical migration and carry out photosynthesis.

Nutrient limitation induces strain-specific physiological changes and emergent migratory traits (see Discussion). Unlike S-1, S-2 cells do not switch to phagotrophy within the observed time window. Furthermore, both swimming speed and orientational stability of S-2 increase as nutrients turn limiting (Fig. 3, B and D, for S-2). The swimming speed of S-2 increased from 89.64 ± 10.25 μm/s to 146.41 ± 30.47 μm/s over the course of 240 hours < *t* < 430 hours, due primarily to the stronger vertical swimming (Fig. 3B and fig. S14). The velocity correlations increase as the nutrient depletion persists, eliciting a significant rise during the late stationary stage (430 hours < *t* < 860 hours), signaling a behavioral shift to a strong ballistic regime (Fig. 3D and fig. S14). This, accompanied by significant increase of orientational stability (reorientation time, τ_r_, reduced from 12.5 ± 5 s to 5.11 ± 2.16 s; Fig. 3E and fig. S24), represents a migratory switch that could allow S-2 population to swim effectively and distribute in the light-rich upper layers. Consistently, S-2 is better adapted to high light conditions, reflected by the lower NPQ values relative to S-1 (Fig. 4 and fig. S21). Although *F*_v_/*F*_m_ and ETR_max_ reduce due to nutrient limitation (*t* test between 336 and 672 hours for both *F*_v_/*F*_m_ and ETR_max_, *P* < 0.001; asterisks indicate significant difference; Fig. 3D, inset), across growth stages the change is lower for S-2 relative to S-1. Together, our results indicate that S-1 up-regulates the light protection mechanism to dissipate excess light energy, whereas S-2 is better light-adapted to maintain their photosynthetic performance. The alteration of migratory properties, in synergy with the photophysiological attributes, enables S-2 to occupy the upper photic layer under nutrient limitation. This effectively broadens the hydrodynamic niche for *H. akashiwo*, thereby maximizing the chances of resource encounters with ephemeral nutrient patches (*20*, *40*).

### Nutrient availability governs strain-specific physiological and behavioral responses

Reconfigurable LDs govern the vertical migration of nutrient-limited raphidophytes, in synergy with strain-specific morphological changes. We have developed a data-based model for cell mechanics to delineate the biomechanical role of LD size and intracellular position on the swimming properties, additionally taking into account the role of nutrient-governed cell morphologies (Materials and Methods, Tables 1 and 2, and supplementary text ST1). When gravitactic swimmers are perturbed to a horizontal position (cell’s long axis perpendicular to gravity vector), S-1 cells in the exponential stage can reorient back and swim against gravity. This is possible when the grey patch where the LDs are localized (computed from Fig. 1C data) lie to the left of the vanishing angular speed (Fig. 4E, dashed line). This confirms the strong ballisticity and low reorientation time (high stability) we observe in the S-1 cells during the exponential stage (Fig. 3, C and E). During late exponential stage, the ballisticity and the reorientation time are reduced because relatively larger LDs are now positioned in the region of vanishing angular speed (grey patch close to the dashed line; Fig. 4F). The angular speed changes to −ve sign with increased magnitude, as shown by the color bar. Overall, cells become neutrally stable, yielding similar probability of turning toward and against the gravity vector. This is experimentally validated by the decreasing swimming speed and ballisticity as the population enters stationary stage (Fig. 3, A and C). Last, cells from the late stationary stage are highly stable in the downward direction (positively gravitactic), due to the large sizes of the LDs and their localization below the nucleus. This results in negative reorientation speeds of ~−0.01 s^−1^, as shown in Fig. 4G and supplementary text ST1 (Materials and Methods). The strain S-1 cells need to expend energy to generate adequate active torque in the opposing direction to swim against gravity, since the grey patch now lies in the region of unstable angular speed (right of the dashed line; Fig. 4, H and I, and supplementary text ST2). LD dynamics, together with the morphological changes, generate biomechanical constraints, thereby affecting the cells’ energetic requirements for sustaining negative gravitaxis.

The increased negative gravitaxis of S-2 under nutrient limitation, in contrast to the behavior of S-1, can be explained by accounting for the morphological changes in our cell mechanics model. As nutrient depletion sets in, S-1 cells preserve their rotational symmetry (Fig. 4B), while S-2 cells become flatter (Fig. 4D), thereby losing the rotational symmetry about their long axis. The ratio between the lengths of the semi-minor (*b*) and semi-major (*a*) axes remains stable for S-1 (*b*/*a* ~0.65 at 60 hours and ~0.6 at 696 hours; Fig. 4, A and B), whereas S-2 cells turn into platelet morphology as represented in Fig. 4 (C and D). The degree of flatness, quantified as (*c*/*a*)^−1^, where 2*c* is the maximum dimension orthogonal to both *a* and *b*, increases from ~1.43 to ~3.26, while *b*/*a* varies from ~0.7 at 96 hours (*36*) to ~0.6 at 860 hours (Materials and Methods). Flattening of the cell shape lowers the active reorientational torque required to maintain negative gravitaxis during the late stationary stage. By mapping *c*/*a* against growing lipid volume (Fig. 4H and Table 3), and the cytoplasmic LD position (Fig. 4I), we compute the active torque required to maintain negative gravitaxis (phase plots in Fig. 4, H and I). An interplay between the cell flatness and the combined effect of LD size and intracellular localization attenuates the active torque required by the S-2 cells to assume a stable gravitactic orientation. The reduction in the active torque follows directly from the drop in the rotational viscous resistance (~30%) experienced by the platelet-shaped cells under prolonged nutrient limitation.

**Table 3.** Parameters used to plot phase diagram of active torque variation in Fig. 4 (H and I).

**Parameter name (symbol)**	**Value**	**Unit**
Major radius (*a*)	7.4	μm
Second major radius (*b*)	4.97	μm
Velocity (*U*)	150	μm/s
Velocity angle (θ)	π/2	rad
Medium viscosity (η)	10^−3^	Pa-s
Density of nucleus (ρ_N_)	1.3	g/cm^3^
Density of lipids (ρ_L_)	0.9	g/cm^3^
Density of cytoplasm (ρ_cyto_)	1.05	g/cm^3^
Density of medium (ρ_fluid_)	1.036	g/cm^3^
Nucleus radius (*r*_N_)	1.94	μm
Nucleus offset along major axis (*L*_N_)	0.37	μm
Lipid volume (*V*_L_)	20	μm^3^
Distance between *C*_L_ and *C*_B_ (*L*_L_)	3.7	μm
Angle between major axis and line joining *C*_L_ and *C*_B_ (φ_L_)	π	rad
Angular velocity (ω)	0.05π	rad/s

In summary, LDs, in synergy with strain-specific morphological shifts, differentially tune the swimming properties under nutrient limitation. The rotational symmetry in cell shape about the cell’s long axis plays a key role in regulating vertical migration. Morphological pliability allows S-2 to harness the spatiotemporal dynamics of cytoplasmic LDs, and thereby enhance the vertical motility. This differential, strain-specific, LD-governed migratory trait is amenable to the measured physiological changes (photosynthesis and trophic strategy) under both nutrient-limited and reincorporated settings (Fig. 4I, inset, and fig. S23, A to C).

## DISCUSSION

### Ecological relevance of strain-specific adaptation under varying nutrient levels

Under nutrient limitation, the species we investigated resort to two distinct strategies, which together indicate their ability to maximize resource acquisition and fitness. Strain S-1 (CCMP3107) reduces vertical motility and shifts to phagotrophy, while strain S-2 (CCMP452) enhances vertical migration, switching to stronger gravitactic behavior. The contrasting strategies emerge because of the active biomechanical control of swimming exerted by the spatiotemporal dynamics of cytoplasmic LDs and strain-dependent morphological pliability. Under persistent nutrient limitation, S-1 would require higher active torque to sustain stable negative gravitaxis (fig. S19) comparable to nutrient-replete conditions. Generation of active torque is an energy-intensive biomechanical process; however, nutrient limitation inhibits its execution. In contrast, the active torque required to maintain the vertical motility attenuates for S-2 (fig. S24) due to the morphological change leading to reduction of the viscous drag. We leverage the experimental and computational data to develop a dissipative power budget to quantify the energy requirement of a swimming cell per unit time, as it gains gravitational energy (due to negative gravitaxis) and undergoes viscous losses (Materials and Methods). We find that the power dissipated reduces for both strains, as cells experience nutrient-depleted environments, thus reducing the active energy requirement by the cells to sustain vertical motility. However, the underlying factors for the reduction of the dissipated power are strain-specific: Strain S-1 suppresses motility and reduces cell size to avoid the viscous dissipation, whereas strain S-2 sustains and even enhances vertical motility, but benefits from a lower viscous energy dissipation due to the morphology-mediated reduction of the rotational and translational drag forces (for S-1 [S-2], the energy dissipation drops from 3.75 to 1.18 fW [4.12 to 2.15 fW]). These biomechanical changes and energetic insights are supported by the commensurate physiological changes, specifically, strain-specific shift in the trophic mode (photo- to phagotrophy of S-1; Fig. 3F) and accompanying strain-specific photophysiological changes (Fig. 3, C and D), ultimately enabling the *H. akashiwo* species to enhance the vertical niche by repositioning across different vertical depths in response to nutrient limitation.

**Fig. 5.**
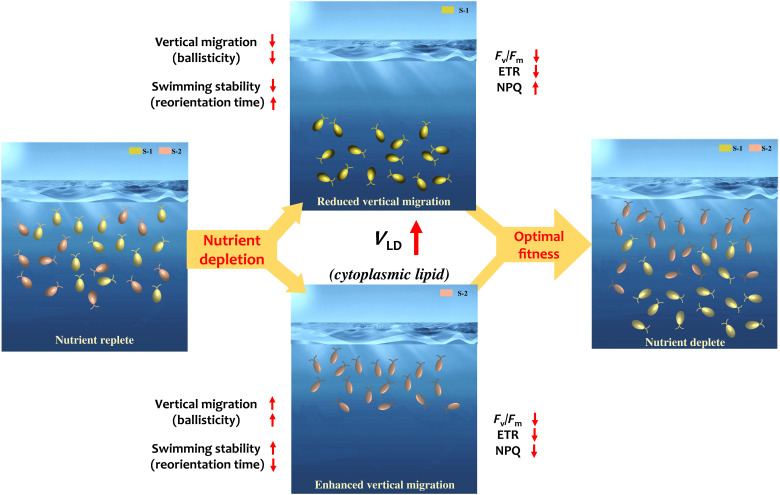
LD-based energy storage and strain-specific migratory shifts act concertedly as coupled traits to enhance fitness under nutrient limitation. Coexisting strains of S-1 and S-2 execute diel vertical migration under nutrient-replete conditions, shuttling between light-rich photic zone during the daytime and nutrient-rich depths at night. While S-1 exhibits stronger motility (high ballisticity and low reorientation time) and higher photosynthetic efficiency (higher *F*_v_/*F*_m_ and ETR_max_) relative to S-2, it is less adapted to high light conditions (low NPQ). Under nutrient limitation, LDs suppress vertical migration of S-1 (swims in deeper water), whereas negative gravitaxis of S-2 strengthens, facilitated by the morphological change. S-1 cells conserve energy by switching from ballistic to diffusive swimming in deeper waters, whereas S-2 cells reduce energy requirements by lowering the viscous losses. The strain-specific behavioral and physiological changes point toward the possibility of concerted yet contrasting adaptive strategies for nutrient acquisition and coexistence (instead of competition) under limited settings.

The strain-specific alteration of *H. akashiwo* swimming due to varying nutrient landscapes can lead to distinct vertical distributions in the laboratory and natural settings (*29*, *30*, *33*, *58*). Although the two strains considered in this study (CCMP3107 and CCMP452) originate from coastal ecosystems, they are genetically distant and exhibit a high degree of interstrain variability (*30*, *33*, *37*). Our results underscore that the shifts in swimming behavior and physiology are coupled traits that could enable bloom-forming species to enhance fitness through coexistence and expansion of the vertical niche under nutrient constraints (Fig. 5 and supplementary text ST3). Under natural settings, high genetic variation is known to facilitate survival of populations under environmental changes by rapid adaptations through selection on different genotypes (*58*, *59*). The dynamic tuning of active torque and dissipative power under nutrient limitation suggests that motile species may undergo exquisite biomechanical adaptations, complementing intrinsic adjustments (*60*). The ability to arrive at a physiologically amenable behavioral trait allows *H. akashiwo* to conserve energy dynamically as nutrient landscapes vary. Sustenance of the vertical motility (in S-2) even under nutrient limitations presents an intriguing yet ecologically meaningful context. Motility not only affects the position of the strain in the vertical niche but also regulates the cell-resource encounter rates, ultimately enhancing the fitness through chanced encounters with ephemeral molecules as and when they are available (*9*, *40*). This is further suggested when we spike the nutrient-depleted cultures with small amounts of fresh nutrients (1 and 5% *C*_0_; inset, Fig. 4I and Materials and Methods). We observe that strain S-1 had a slower growth than strain S-2, although under replete conditions S-1 has a higher growth rate. This indicates that, under nutrient-limited conditions, S-2 has a potentially higher nutrient affinity than S-1, i.e., better ability to use miniscule amounts of nutrients as and when they are available. Together with this, the observation of a relatively more rapid lipolysis in S-2 relative to S-1 (Fig. 2), our results demonstrate that *H. akashiwo* harness contrasting interstrain traits to maintain a viable population via expansion of the vertical niche and diversification of the trophic strategies toward securing available energy resources (Fig. 5 and supplementary discussions) under nutrient-limited conditions (*58*, *61*). Confirming earlier reports that the vertical distribution of *H. akashiwo* is strain-specific (*30*, *33*), our results shed light on emergent adaptations that enable *H. akashiwo* to explore distinct motility and trophic strategies along the vertical column. Although the current study is based on the raphidophyte *H. akashiwo*, our results on LD-governed biomechanics of motility are generic and applicable for characterizing the swimming and buoyancy regulation in diverse phytoplankton taxa, including diatoms and dinoflagellates, under nutrient limitation. Further work is needed to analyze whether and how phytoplankton species could exploit LD translocation to vertically redistribute their population, particularly in response to environmental changes (*59*, *61*), allowing extension of their vertical niches and diversification of the behavioral strategies to adjust to the emerging resource landscapes.

Diversification of traits, both physiological and behavioral, enhances the chances of survival of species across a wide range of environmental settings (*36*, *53*). Understanding how phytoplankton adapt and survive the rapidly evolving nutrient landscapes of today’s oceans remains a crucial challenge. Accurate prediction of harmful blooms due to the raphidophytes, and more broadly the cascading biogeochemical implications of phytoplankton adaptations, will rely on mechanistic understanding of coemerging behavioral and physiological responses, and the interrelations therein (*2*, *62*). Our data suggest that migration and nutrient storage—so far considered independent traits (*40*)—coevolve in a concerted fashion to expand the vertical niche and diversify the trophic strategy, thereby maximizing the chances of survival of *H. akashiwo* under nutrient-limited settings. Extending beyond raphidophytes, our results may provide new mechanistic insights into the paradoxical diversification of phytoplankton species in general (*63*, *64*), offering a fresh perspective to the coemerging adaptive traits in phytoplankton under stressful environments. This will open up quantitative avenues to assess impacts of multiple stressors (*1*) on phytoplankton, beyond and in conjunction with the evolving nutrient landscapes of today’s oceans.

## MATERIALS AND METHODS

### Cell culture

We focus on two different strains of raphidophyte *H. akashiwo*, namely, CCMP3107 (S-1) and CCMP452 (S-2), for our investigations. For the experiments, cells were cultured in 50-ml sterile glass tubes under a diel light cycle (14-hour light:10-hour dark) in f/2 (minus silica, −Si) medium (*C*_0_) at 22°C. For propagation of the cell cultures, 2 ml of the parent culture was inoculated into 25 ml of fresh medium every 2 weeks. For propagation of cultures in nutrient-limited environments, f/2 (−Si) medium was appropriately diluted in artificial sea water (ASW; 38 g/liter of sea salts in MilliQ water) to reach 10% *C*_0_ [10× dilution, 10 ml of f/2(−Si) and 90 ml of ASW] and 1% *C*_0_ [100× dilution, 10 ml of 10× f/2(−Si) and 90 ml of ASW] dilutions. S-1 and S-2 cultures used for phenotypic trait quantification experiments were propagated from a 5- to 7-day-old preculture (mid-exponential growth stage) to standardize the starting population physiological status. A fixed period of the day (between 9:00 and 15:00 hours) was chosen for the experiments to rule out any possible impact due to the diurnal migration pattern of phytoplankton *H. akashiwo*. Here, we note that for the population-scale motility analysis, we have generally used three biological replicates, each of which is complemented with three technical replicates, unless otherwise specified.

### Quantification of LD volume and intracellular effective size and localization

To characterize and quantify the biosynthesis and accumulation of LD, cells were sampled from the culture tube at different time intervals to cover whole exponential and stationary growth stages and stained with neutral lipids fluorescent stain Nile Red (Thermo Fisher Scientific; excitation/emission, 552/636 nm). Ten microliters of 100 μM Nile Red [in dimethyl sulfoxide (DMSO)] was dissolved into 200 μl of *H*. *akashiwo* culture supernatant and mixed thoroughly by vortexing the mixture. Cells (200 μl) were added to the mixed NR stain aliquot (final concentration, 2.4 μM) and incubated in the dark at room temperature for 15 min. To identify and characterize the accumulation of LDs in single cells, we used phase-contrast and fluorescence microscopy (Olympus CKX53 inverted microscope) supplemented with high-resolution color camera (Imaging Source, DFK33UX265). To avoid any photo-toxicity effect of light during cell analysis, excitation light-emitting diode (LED) intensity (552 nm) was kept low and at a maximum value of 5. To extract the S-1 cell area and both LD dimension (area and volume) and intracellular location, movies of minimum of 20 representative single cells were recorded at 16 frames per second for 5 s. We quantified the LD effective size assuming individual LDs to be a spherical droplet and deriving the effective radius of individual LDs from the contour area extracted by thresholding the images using in-house MATLAB image processing algorithms and ImageJ. From the individual LDs, the position of their net center of mass in single cells was determined with respect to the cell geometric center.

### Quantification of nucleus radius and intracellular localization

To determine nucleus size and position, S-1 cells were sampled from the culture tube at different time intervals representative of lag (60 hours), exponential (300 hours), and stationary (700 hours) growth stages and stained with Syto9 Green Fluorescent Nucleic Acid Stain (Thermo Fisher Scientific; excitation/emission, 480/501 nm). A total of 7.5 μl of 100 μM Syto9 (in DMSO) was dissolved into 300 μl of S-1 cell supernatant and mixed thoroughly by vortexing. Cells (300 μl) were added to the mixed Syto9 stain aliquot (final concentration, 1.2 μM) and incubated in the dark at room temperature for 12 min. To quantify nucleus characteristics in single-cell S-1, we used phase-contrast and fluorescence microscopy (Olympus CKX53 inverted microscope) supplemented with high-resolution color camera (Imaging Source, DFK33UX265). To avoid any photo-toxic effect of light during cell imaging, LED excitation intensity (480 nm) was kept low and at a maximum value of 5. To extract S-1 nucleus radius and intracellular localization, movies of minimum 20 representative single cells were recorded at 16 frames per second for 5 s. We derived the effective radius of the nucleus from the contour area extracted by thresholding and ImageJ image analysis. Relevant data on S-2 can be found in (*36*, *53*). The analysis of the cell morphology, lipids, and cell nucleus (all measurements in micrometers) uses weighted mean and weighted SD to estimate their statistics.

### Reorientation analysis in the dynamic experimental setup

The swimming stability of S-1 was quantified for two time points representative of exponential (72 hours) and stationary (700 hours) growth stages. Experiments were conducted in rectangular millifluidic chamber (10 mm × 3.3 mm × 2 mm) made of polymethyl methacrylate with ~1.6-mm inlet and outlet circular holes located at the edges of chamber opposite to each other (see fig. S15). The chamber was mounted via magnets on a circular plate, which is, in turn, attached to a custom-made holding cage that includes light diffuser (220 grits) and a red LED (630 nm wavelength). The different components in the cage are placed in a way to permit homogeneous lit of the interested chamber area. One end of the entire cage is connected to the shaft of a stepper motor that permits the rotation of the whole setup as needed. The Nema 23 stepper motor was controlled automatically using a DM452T driver coupled to an Arduino uno board. The automation part comes from programming the Arduino to rotate by a specific degree for a specific duration. On the other end of the cage, a variable zoom lens system (zoom = 1.7×) was connected to a Grasshopper3 (model: GS3-U3-41C6C-C) camera with a 1″ sensor that permits to capture the entire vertical height of the chamber. The geometric center of the chamber, light source, and diffuser were placed in coaxial position. For imaging, the focal plane was chosen visually to be in the middle of the experimental millifluidic chamber.

Before image acquisition, concentration of cells in the measured sample was adapted to permit single-cell tracking and quantification of corresponding reorientational stability (*36*). Because of low cell density, no dilution was required at 72 hours. During late stationary stages, the cell cultures were diluted in a glass vial supplemented with 3.4 ml of 0.45-μm filtered culture supernatant collected from the bottom of the experimental culture tube using a 120-mm needle and 2-ml syringe. Cell culture (1.2 ml) collected from the top 5-mm tube were gently added in the filtered supernatant volume to reach a 3.8× final dilution. The glass vial with the cell dilution was covered with parafilm with multiple holes done using small needle to allow airflow and placed in the incubator at 22°C for 50 min to allow cell acclimatization. We conducted two biological replicates with three technical and three technical replicates, numbering a total of 18 replicates for each of the two strains analyzed. Millifluidic chamber was filled by gently pipetting suspension of phytoplankton cells (~180 μl) sampled from the top 0.5-cm culture followed by closing the inlet and outlet ports with a silicone plug and mounting it on the magnetic cage. A wait time of 20 min was given so that cells distributed in the most stable configuration in the millifluidic chamber. Afterward, a 180° rotation was applied to the chamber through the Arduino controlled automatic stepper motor (programmed in-house). Once cells swam to the middle of the chamber, we applied a series of three consecutive flips with intervals of 15, 12, and 12 s for cells at 72 hours and 25, 20, and 20 s for cells at 700 hours. The different waiting periods were adapted considering the growth stage–dependent reorientation speeds (speed at stationary stage is lower than speed at exponential stage; Fig. 3). Videos were acquired at 16 frames per second. Phytoplankton cells were tracked using ImageJ plugin Mosaic Particle Tracker (2D/3D) and analyzed using in-house Python codes. We analyzed 160 and 220 frames for populations at 72 and 700 hours, respectively. The corresponding swimming times of 10 and 14 s were sufficient to capture multiple cell reorientation events in each replicate. Among the acquired trajectories, those that appear for less than 45 frames or those that showed a net displacement less than 10 pixels (corresponding to 20 μm, one cell body length) were eliminated. The remaining trajectories were interpolated quadratically (smoothing). For single trajectory, angular velocity (ω) was obtained for each consecutive frame as a function of the instantaneous angular position (θ). Angular velocities were averaged for given θ value (ranging from −90° to 90°, binned at intervals of 10°). Any ω value greater than 0.5 rad/s or less than −0.5 rad/s was eliminated. Obtained ω values were fitted to a sinusoidal curve of the form *A* × sin(*x*), and the reorientation time scale was then obtained as $B=\frac{1}{2A}$.

For calculation of S-2 reorientational stability, similar setup was used. Young cells were 96 hours (three biological replicates with two technical replicates each), and old cells were between 720 hours (one biological replicate with one technical replicate) and 1080 hours (two biological replicates with two technical replicates each). Old cells were diluted like S-1. Similar image acquisition, processing, and data analysis pipeline were applied. Images were acquired at 4× zoom for the two bioreplicates and 1.1× for the single replicate. Angles were binned at an interval of 10°. Among the acquired trajectories, those that appear for less than 45 frames for 4× zoomed and less than 20 frames for 1.1× zoomed or that showed a net displacement less than 30 pixels for 4× zoomed images or 10 pixels for 1.1× zoomed images were eliminated.

### Determination of motility phenotypes by static flipping chamber experiment

An adapted setup of the one previously described was used to characterize the cell motility phenotypes. Experiments were conducted in a millifluidic chamber (12 mm × 4 mm × 1.6 mm) constructed out of an acrylic sheet and mounted on a supporting frame. To homogenize the culture before the experiment, culture tube was gently mixed by gently rotating the tube of 360° for two times. Suspensions of phytoplankton cells (~180 μl) sampled from the top 0.5-cm culture were gently pipetted into the chamber through one of the two injection ports. The flipping chamber was mounted on a translational stage, the position of which could be controlled using micrometer screws along all three axes. During experiments, cells in the flipping chamber were visualized using an optical element (Navitar, 1-60191 AD, 6.5× Ultra Zoom, 12 mm FF) and a digital camera (Imaging Source DFK33UX265). The suspension was uniformly illuminated using a red LED (630 nm) mounted outside of the flipping chamber. After the phytoplankton population reached its equilibrium distribution over the vertical of the chamber (~10 min), we rotated manually the chamber for 180° through its perpendicular plane. After chamber flipping, images were acquired in the midchamber plan of focus at 16 frames per second for 30 s.

### Cell tracking

To extract the swimming behavior of phytoplankton cells, movies were recorded at 16 frames per second for 30 s. For tracking, cell locations were determined by image analysis based on intensity thresholding using ImageJ routine. ImageJ plugin Particle Tracker 2D/3D was used to assemble cell trajectories by linking the locations of cells in subsequent frames and obtain the coordinates (geometric center) of the cells at each interval. Single-cell coordinate at each interval was used to extract trajectories of individual cells and quantify the respective swimming speed, horizontal component, and vertical component velocity. For swimming speed analysis, we considered only trajectories longer than 100 frames (corresponding to a minimum of 6 s of swimming).

### Pulse-amplitude modulated chlorophyll fluorometry experiment

Pulse-amplitude modulated chlorophyll fluorometry (PAM) was used to evaluate and quantify the photophysiological efficiency of *H*. *akashiwo* cells at different time intervals during the exponential and stationary growth stages in diverse nutrient regimes. Multiple Excitation Wavelength Chlorophyll Fluorometer (Multi-Color-PAM; Heinz Walz GmbH, Effeltrich, Germany) was used to quantify the maximum photosynthetic quantum yield (*F*_v_/*F*_m_), the maximum electron transport rate (ETR_max_), and NPQ of *H*. *akashiwo* cells at the population scale. For PAM measurements, culture tube was mixed by gently rotating the tube by 360°. Suspensions (1200 μl) of plankton cells were sampled from the top 0.5-cm culture and placed into a quartz silica cuvette (Hellma absorption cuvettes; spectral range, 200 to 2500 nm; pathlength, 10 mm). We used Multi-Color PAM 3 Win software saturation pulse (SP) and light curve method to quantify *F*_v_/*F*_m_ and ETR_max_ at diverse time intervals and under different nutrient regimes. We have taken two biological replicates and complemented with two technical replicates from each for the experiments.

### Quantification of endogenous cellular stress

Endogenous ROS were quantified with the fluorescent stain CellROX Orange (Thermo Fisher Scientific; excitation/emission, 545/565 nm) to determine impact of nutrient limitation on ROS production. CellROX Orange is a cell-permeable reagent nonfluorescent while in a reduced state and upon oxidation exhibits strong fluorogenic signal. *H*. *akashiwo* cells sampled at different nutrient regimes were incubated with 5 μM CellROX Orange for 30 min in the dark (400 μl of cells with 0.6 μl of CellROX ready-to-use stock solution). After incubation, cells were illuminated using green light (~545 nm) with an exposure time of 1/5 and an LED fluorescence intensity of 100%. The fluorescence readout was quantified over 21 s using fluorescence microscopy (Olympus CKX53 inverted microscope) supplemented with high-resolution color camera (Imaging Source, DFK33UX265). For single cell, the fluorescence intensity is increased over time; the magnitude of fluorescence intensity before cell lysis corresponded to the maximum ROS accumulation in the cell. For quantification, acquired single-cell fluorescence image was analyzed with ImageJ Z-stack layer to extract time-dependent signal intensity variation. Stress accumulation rate, quantified as fluorescence intensity signal, was integrated over the first 10 s of acquisition.

### Nutrient-starved phytoplankton biomechanical and physiological response to fresh nutrients

Nutrient-depleted *H*. *akashiwo* cells from the stationary growth stage exhibit characteristic biomechanical and physiological properties (LD accumulation, motility, and reduced photophysiology), which enable phytoplankton population to save energy and survive nutrient constraints. Cultures of *H*. *akashiwo* cells from stationary growth stage (700 hours) were supplemented with low amount of fresh f/2(−Si) to evaluate and quantify phytoplankton biomechanical and physiological recovery efficiency during a short time interval of 48 hours. Eight milliliters of late stationary cell cultures was reincorporated with (i) 1 ml of *C*_0_ f/2(−Si), (ii) 1 ml of 10% *C*_0_ f/2(−Si), and (iii) control (no nutrient reincorporation). Total nutrient (nitrate and phosphate) concentrations per cell (quantified to stationary cell concentration of 10^5^ cells ml^−1^) were estimated to be NO_3_^−^ (i) ~7 × 10^−7^ μmol cell^−1^, (ii) ~7 × 10^−8^ μmol cell^−1^, (iii) 0 μmol cell^−1^; PO_4_^3−^ (i) ~4.5 × 10^−7^ μmol cell^−1^, (ii) ~4.5 × 10^−8^ μmol cell^−1^, (iii) 0 μmol cell^−1^. After 24 hours of incubation at 22°C (14 hours light,10 hours dark), *F*_v_/*F*_m_, ETR_max_, total LD volume per cell, motility, and ROS were quantified (as described previously) to determine recuperation of *H*. *akashiwo* motility and physiological recovery efficiency.

### Quantification of nutrient concentration

Analysis of nutrients, namely, nitrate (NO_3_^−^) and phosphate (PO_4_^3−^), were performed colorimetrically using Prove600 Spectroquant (Merck). NO_3_^−^ was analyzed with Nitrate Cell Test in Seawater (method: photometric 0.4 to 13.3 mg/liter NO_3_^−^ Spectroquant). The corresponding detection limit of NO_3_^−^ concentration measurable in our experiments is 6.45 μM. PO_4_^3−^ was analyzed with phosphate cell test (method: photometric 0.2 to 15.3 mg/liter PO_4_^3−^ Spectroquant, with corresponding detection limit of 2 μM). Experimental values below the respective detection limits are taken as zero.

### Statistical analysis

We performed one-way ANOVA to compare the total LD volume accumulation in single S-1 among different time intervals from exponential to stationary growth stage. We made multiple comparisons using a post hoc Tukey’s honest significant difference test. Same multiple comparison statistical analysis was conducted to compare phytoplankton speed and photophysiological parameters among different time interval samples representative of the exponential and stationary growth stages. Same statistical analysis was conducted in the nutrient recovery experiment to compare photophysiological efficiency, LD volume, motility, and ROS among the still (control, no nutrients) and cells supplemented with fresh f/2(−Si) after 24 hours of incubation. Statistical analysis figure wise:

Figure 1B: ANOVA: *P* < 0.001; asterisk indicates significant difference in *V*_LD_ between *t* = 228 hours and *t* = 300 hours. Figure 1F: *t* test between 696 and 1080 hours and one-way ANOVA between 288, 696, and 1080 hours, *P* < 0.001; asterisks indicate statistically significant difference. Figure 2B: One-way ANOVA between 10, 18, and 24 hours, *t* test between 34 and 42 hours, *P* < 0.001; asterisks indicate statistically significant difference. Figure 2G: One-way ANOVA between 12, 24, and 36 hours, *t* test between 24 and 36 hours, *P* < 0.001; asterisks indicate statistically significant difference. Figure 3A: The significant differences (one-way ANOVA, *P* < 0.001, post hoc Tukey’s honest significant difference) between the exponential (*t* = 100 and 200 hours), late exponential (*t* = 250 hours), and stationary (*t* = 400 and 700 hours) growth stages are observed. The bar plots represent the mean swimming speed ± SD; one asterisk indicates statistically significant difference to 100 and 200 hours; at 400 hours, two asterisks indicate statistically significant difference relative to swimming speeds at 100, 200, and 250 hours. At 700 hours, three asterisks indicate statistically significant difference to 100, 200, 250, and 400 hours. Figure 3C (inset): *t* test between 324 and 540 hours for both *F*_v_/*F*_m_ and ETR_max_ *P* < 0.001; asterisks indicate statistically significant difference. Figure 3E: The bar plot represents mean ± SD, and the asterisk indicates statistical significance between the reorientation time of exponential and stationary stages (two-sample *t* test, *P* < 0.001). Figure 3B: One-way ANOVA between 96, 168, 240, and 430 hours, *t* test between 240 and 430 hours, *P* < 0.001; asterisks indicate statistically significant difference. Figure 3D (inset): *t* test between 336 and 672 hours for both *F*_v_/*F*_m_ and ETR_max_, *P* < 0.001; asterisks indicate statistically significant difference.

### Mean squared displacement estimation

Quantification of ballistic motility is ensured through a rigorous cell tracking followed by mean squared displacement (MSD) analysis. For the analysis of MSD and the corresponding velocity correlations (insights and details in the main draft), we closely follow the procedures in (*65*). The package @msdanalyzer is modified and used in MATLAB to obtain the MSD curves for 60, 300, and 700 hours culture age (curves shown in fig. S14). The log-log representation of the MSD plots can be fitted with a linear function, that is, log*(<r*^2^*>) =* Γ *+* αlog(*t*), where the exponent α provides the information whether the flow is diffusive (α ~ 1), ballistic motion/active transport/super-diffusive (α ~ 1.5 to 2), or constrained transport (α < 0.9) in nature. To obtain the exponents, we clip the MSD plots until where the phytoplankton cells do not feel the confinement effect. At large times, the confinement effect will always be felt and the confinement signature is reflected in the MSD plots.

### Cell mechanics model and phase plot

To understand the cell stability, dead and active torque, and cell reorientation time scale (as obtained from experiments), we propose a reduced-order model for the cell mechanics. Here, we talk in terms of force and torque balance on the phytoplankton cell and attempt to decipher the effect of lipid formation on the cell stability and rotational kinetics. The focus of the discussion is to identify the relevant forces and torques a motile cell would encounter. A cell experiences a propulsion (***P***) attributed to the drive from its flagella, the weight of its own body that can be segregated into weight from its nucleus, lipids, and cytoplasm, an upthrust due to its density being not equal to the surrounding fluid and a drag force attributed to the viscous effects. The relevant torques about its center of buoyancy will include one from nucleus and lipids (if they do not reside on the major axis of the cell), torque due to viscous drag, and torque induced due to translational drag for asymmetric cells (*36*). With these considerations, we formulate the overall force and torque balance in each component as follows (see fig. S10)

$$\begin{array}{c} P\text{sin}\varphi =D\text{sin}\theta \\ P\text{cos}\varphi -D\text{cos}\theta =(\rho_{\text{cyt}}-\rho_{\text{fluid}})V_{C}g+(\rho_{N}-\rho_{\text{cyt}})V_{N}g+(\rho_{L}-\rho_{\text{cyt}})V_{L}g\\ D\text{sin}(\theta -\varphi )L_{H}-W_{N}\text{sin}(\varphi_{N})L_{N}-W_{L}\text{sin}(\varphi -\varphi_{L})L_{L}=R\eta \omega \end{array}$$ (1)

Here, ρ, *L*, *V*, and η denotes the density, distance from cell centroid, volume, and medium viscosity, respectively. The subscripts C, cyt, fluid, N, L, and H refers to the cell, cytoplasm, background medium, nucleus, lipids, and the hydrodynamic center of the cell, respectively. φ_N_ is the angle between the direction of gravity (vertical line) and the line joining *C*_N_ and *C*_B_, while φ_L_ is the angle between the major axis and the line joining *C*_L_ and *C*_B_. φ is the angle of the cell propulsion axis (resultant flagellar motion) and the vertical axis, which is an unknown along with the propulsion force **P**. These definitions of the angles make them independent of the initial angular position of the cell and dependent on φ, which comes as part of Eq. 1. Because we assume the center of gravity of the nucleus to lie on the major axis, an assumption supported experimentally, therefore, φ_N_ = φ. Realistically, a cell does not move exactly in the direction of propulsion and is assumed to move at an offset making an angle θ, a known parameter obtainable from experiments, with the vertical. In the above configuration, counterclockwise angular direction from the vertical line (direction of gravity) is assumed to be positive. The terms *W*_L_ and *W*_N_ are the weight of the lipids and the nucleus with respect to the cytoplasm. *D* is the drag force described below, *P* is the force of propulsion that is unknown and is obtained from solving Eq. 1. For the sake of brevity, we have neglected the fore-aft asymmetry of the cellular structure, thereby putting forward the central message without sacrificing the essential physics. Nevertheless, in the supplementary discussion, we have highlighted that, accounting for the fore-aft asymmetry of the cells, the present trend will be reinforced. The cell is described by the generic equation $r=\frac{\mathrm{abc}}{\sqrt{c^{2}(b^{2}{\text{cos}}^{2}\gamma +a^{2}{\text{sin}}^{2}\gamma ){\text{cos}}^{2}\psi +\alpha^{2}b^{2}{\text{sin}}^{2}\psi }}$ , where *a, b* (*a > b*), *c (=b)*, γ (0 < γ < 2π), and ψ (−π/2 < ψ < π/2) denote the major axis length, semi-major axis length, minor axis length, azimuth angle, and polar angle, respectively. The symmetric geometry implies that the hydrodynamic center of the cell is on the cell centroid (center of buoyancy) and *L*_H_ vanishes (*36*). Last, *R* represents the coefficient of hydrodynamic rotational resistance on the phytoplankton cell. To obtain the cell dimensions, we have fitted the phase-contrast microscopy images to the cell profile using the above equation and imposing γ = 0, thereby obtaining a 2D version of the parametric equation of the form $r=\frac{\mathrm{ab}}{\sqrt{b^{2}{\text{cos}}^{2}\psi +\alpha^{2}{\text{sin}}^{2}\psi }}$ (*66*). Because the cell is assumed symmetric prolate ellipsoid, the drag can be expressed as *D*_∥, ⊥_ = 6πη*r*_eq_*UK*_∥,⊥_, where *U* is the translational velocity and *K* denotes the shape factor having the form $K_{∥}=\frac{4{\left(t^{2}-1\right)}^{3/2}}{3t^{1/3}\{(2t^{2}-1)\text{ln}[t+{\left(t^{2}-1\right)}^{1/2}]-t{\left(t^{2}-1\right)}^{1/2}\}}$ and $K_{⊥}=\frac{8{\left(t^{2}-1\right)}^{3/2}}{3t^{1/3}\{(2t^{2}-3)\text{ln}[t+{\left(t^{2}-1\right)}^{1/2}]+t{\left(t^{2}-1\right)}^{1/2}\}}$ for prolate spheroids (*67*, *68*) with *t = a*/*b*. The coefficient of resistance has the form $R=C_{R}\frac{2(t^{2}+1){\left(t^{2}-1\right)}^{3/2}}{3t\{(2t^{2}-1)\text{ln}[t+{\left(t^{2}-1\right)}^{1/2}]-t{\left(t^{2}-1\right)}^{1/2}\}}$ (*67*), with *t* holding the same meaning as above and $C_{R}=8\pi r_{\text{eq}}^{3}$. The viscous torque drag can be estimated using τ = *R*ηω, where ω is the angular velocity. The meaning of the rest of the symbols is explained in the above model geometry. This set of three equations has three unknowns, namely, ***P***, **ϕ**, and **ω**. The solution we are interested is in finding the angular rotation rate **ω**. Thus, from all the values known from the experiments across different growth stages, we attempt to draw a phase plot (represented in Fig. 4, E and G) that reflects the value of the angular rotation rate as a function of the different possible positions (varying φ_L_ and *L*_L_) a representative LD of dimensions corresponding to a particular growth stage can take within a cell. The phase plots highlight the stability of the cell emerging from various factors. With nucleus remaining very close to the geometric center, the dominant factor to impart the stability criterion to the strain S-1 cells is the lipid compartmentalization effects.

To estimate the active torque for strain S-2, we must acknowledge that the translational and rotational viscous resistance must vary with the cell shape. Toward accounting for this, we use the following semi-analytical formulation to obtain the cell viscous resistance. The expression for the active torque is given by $\tau_{\text{ac}}=W_{N}\text{sin}(\phi_{S})L_{N}+W_{L}\text{sin}(\phi_{L})L_{L}+\tilde{R}\eta \omega$, where $\tilde{R}$ is given below.

For any general ellipsoid, the drag force *D* applied by an arbitrary ellipsoid, with semi-axis lengths {k, m, n}, on the fluid when translating at speed *U* in the *k* direction, is $\frac{D}{\pi \eta U}=\frac{16}{\phi +\zeta_{k}k^{2}}$, while the torque applied on a fluid due to rotation with rate ω around the *k* direction is $\tilde{R}=\frac{\tau }{\pi \eta \omega }=\frac{16}{3}\frac{m^{2}+n^{2}}{m^{2}\zeta_{m}+n^{2}\zeta_{n}}$ (*69*), where the variables in the relation are given by $\zeta_{k,m,n}=\int_{0}^{\infty }{\mathrm{dx}}^{'}\frac{1}{({\left(k,m,n\right)}^{2}+x^{'})\sqrt{\left(k^{2}+x^{'})(m^{2}+x^{'})(n^{2}+x^{'}\right)}}$ (with *k*, *m*, and *n* denoting individual relations for ζ*_k_*, ζ*_m_*, and ζ*_n_*, respectively) and $\phi =\int_{0}^{\infty }{\mathrm{dx}}^{'}\frac{1}{\sqrt{\left(k^{2}+x^{'})(m^{2}+x^{'})(n^{2}+x^{'}\right)}}$. Note that we have used $\tilde{R}$ instead of R to distinguish it from the torque resistance for strain S-1 cells, albeit these are equal when both the semi-minor axes of the cell equate. We have estimated the integrals using MATLAB and noting that the integral is sensitive to initial discretization of *x’*, and a log-spaced vector is used for accurate integral estimation. We have obtained an error of <10^−4^% between analytical *R* and numerical $\tilde{R}$ values during comparison. The active torque is plotted as a function of different ratios of the shortest axis (*c*) and major axis (*a*), different ratios of the lipid volume by cell volume, and different ratios of the lipid distance from the cell geometric center to major axis of the cell. All other factors for strain S-1 and S-2 are kept the same for comparison of the required active torque. To obtain the active torque, we have used the tabulated data as given here (except for the axes in the phase plots that were varied in Fig. 4, H and I).

### Flagellar dynamics experiment

For each growth stage, five cells were randomly chosen for analysis. For each cell, the first 150 frames were taken. Starting from the first frame, the flagellum was observed manually until a sinusoidal pattern was observed (half-wave or full wave). The amplitude (*A*) and wavelength (λ) is measured using ImageJ. The frame number is noted. Then, subsequent frames are observed until a similar pattern (stroke) emerged and same measurements are taken, along with the new frame number. The difference between consecutive frame numbers (nf) is noted. This process is repeated for six strokes. The final amplitude (*A*_av_) and wavelength (λ_av_) are averaged over these six values. The final frequency (*f*_fin_) is calculated as fps/nf_av_, where nf_av_ is the average different between consecutive frame numbers. These values are then plugged into the formula *E*, where *E*_s_ is the specific energy defined as energy per unit μ, with μ being the mass of the flagella per unit length. Note that ω = 2π*f*_fin_. The specific energy from five cells is averaged for each growth stage, and the final ratio is obtained. While these are gravitactic organisms, microscopy was done in the horizontal plane, but, for both growth stages, effectively negating any potential alteration in behavior.

## Supplementary Materials

### This PDF file includes:

Supplementary text (ST1 to ST3) Figs. S1 to S24 References
