# Supplementary material for: Active reconfiguration of cytoplasmic lipid droplets governs migration of nutrient-limited phytoplankton
Source: Sci Adv. 2022 Nov 4;8(44):eabn6005. doi: 10.1126/sciadv.abn6005 (PMC11633079; doi:10.1126/sciadv.abn6005)
Supplement: Supplementary file 1 — Supplementary text (ST1 to ST3) Figs. S1 to S24 References [file sciadv.abn6005_sm.pdf]

Science Advances
AAAS

Supplementary Materials for

Active reconfiguration of cytoplasmic lipid droplets governs migration of nutrient-limited phytoplankton

Anupam Sengupta *et al.*

Corresponding author: Anupam Sengupta, anupam.sengupta@uni.lu

Sci. Adv. **8**, eabn6005 (2022)
DOI: 10.1126/sciadv.abn6005

This PDF file includes:

Supplementary text (ST1 to ST3)
Figs. S1 to S24
References

Supplementary Text

ST1. Cell fore-aft asymmetry: During the estimation of the active torque, we have excluded the estimation of the torque induced due to the fore-aft asymmetry of the cellular morphology. This torque is quantified using the expression $D\sin(\theta-\varphi)L_H$ as given in equation 1 (Methods) where D is the drag on the cell and L_H is the distance between the cell's centre of buoyancy and centre of hydrodynamic stress. The value of L_H can be estimated from hydrodynamic simulations as performed previously and depends on the fore-aft asymmetry parameter d_{fa} in the relation $r=\frac{ab}{\sqrt{b^2\cos^2\psi+\alpha^2\sin^2\psi}}+d_{fa}\cos\psi$ (*70*). Here we discuss how the fore-aft asymmetry affect the dynamics and does it reinforce or alters our prediction from the mechanistic point of view.

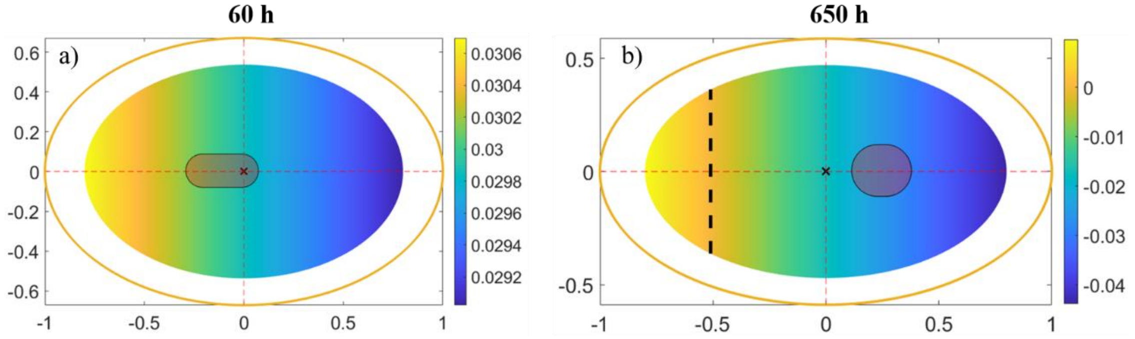

Fig. S1. Phase plots in consideration of fore-aft asymmetry morphology. We observe that accounting for fore-aft asymmetry in S-1 reinforce the findings we have in Figure 4. The exponential cells become highly stable implying a stong balistic motion while stationary cells are high unstable, showcasing their reduced balisticity.

For S-1, we observe that there occurs a slight fore-aft asymmetry at their exponential stage. We find the value of d_{fa}/a close to 0.12 as also observed previously (*36*). In sharp contrast, we observe that (Figure S4) that the cells either lose their fore-aft asymmetry becoming eliptical in morphology or gain a slight reverse fore-aft asymmetry. In both these cases, the cell therefore, tend to become more unstable. Considering a value d_{fa}/a half of its exponential value as ~ – 0.055 (minus sign signifying the reverse fore-aft asymmetry), we find from the phase diagram (Figure S1) that the cells have a default re-orientation towards gravity. Therefore, consideration of fore-aft asymmetry conforms to and reinforces the predictions made by the biomechanics model.

For S-2, we observe that d_{fa}/a close to 0.13 at their exponential stage. This lends to some stability of exponential cells against their top heavy nature of the nucleus; nevertheless the cell would remain unstable. At stationary stage, however, these cells maintain their fore-aft asymmetry, unlike S-1. Furthermore, these cells exhibit a higher linear velocity but a flat morphology, overall keeping the drag and thereby the torque due to fore-aft asymmetry similar to that of its exponential stage cells. Overall, fore-aft asymmetry does not play a major role across the growth stage for S-2 in altering the predictions made by our biomechanics model.

ST2. Flagellar dynamics in viscoelastic fluids and how it can affect to further reduce the cells viscous behavior: (a) Mechanical Energy Budget and Flagella Beating. One primary message that was highlighted from the experimental observation was the definitive strategy of the phytoplankton cell to decrease their overall energy consumption. Here we attempt to build upon this aspect with somewhat quantitative manner through a mechanical energy expense budget of a motile cell in its strive towards moving in their favorable direction, *i.e.* up-swimming. As a follow up, a qualitative discussion will follow in context of flagella beating of a cell discussing the energy expense at the source. The main aim in this short discussion is to present the idea that

the cell attempts a conscious reduction in its mechanical energy expense as it goes from exponential growth stage into its stationary growth stage. While a congruent argument will be postulated to unite all the emerging characteristics, this study has reported. It is, however, important to acknowledge that all the involved energies in cellular dynamics, broadly mechanical, biochemical, photosynthetic and associated energy losses, are not accurately measurable and hence our analysis target the cell's ability to reduce the mechanical energy expense and maneuver their motility characteristics to their advantage. Towards this, we identify the collective mechanical energy that is spent on a cell body to maintain its basic motile behavior. These energies include the potential energy due to rise, the viscous dissipation energy loss due to translation and due to rotation. The power expense for potential energy rise, translation and rotational viscous loss has, respectively, the form mgU_y, $D\boldsymbol{U}$ and $R\eta\omega^2$ (*72*), where U_y is the y-component of the velocity field, m is the resultant cell mass (considering nucleus, lipids and cytoplasm) and all other symbols carry the same meaning as described in the cell mechanics model, D is the translation drag force and R is the rotational drag torque (note for solving the energy budget in case of strain S-2, R is replaced by R). The expression for drag and torque for the ellipsoids are detailed in the cell mechanics model section. From the above value, we see that $\boldsymbol{U}$, U_y and ω are variables that depend on motile characteristics of the cell while the drag and torque coefficients depend on the cell morphology. Both motility and morphology vary across the growth stages and thus, we have considered these variations from experimental observations. Plugging in the actual experimental values at the corresponding time points, we obtain the mechanical power loss for S-1 in early exponential stage (60 h) is ~3.75fW while that in the stationary stage (700 h) is ~1.18 fW. Therefore, a clear situation arises where the cell attempts to save on its mechanical energy expenditure, which has intriguing consequences on its survival strategy, as has been discussed in the main text. Note that, theoretically the rate at which energy is expended per period of generic flagellum motion (detailed in next section) comes in order of 10^{-13}W while a conversion efficiency to swimming remains less than 8% (*74*). Interestingly these values come close to the overall mechanical power in orders of fW exhibited by the phytoplankton motion, as described in this section. For S-2, we employ a similar analysis, we observe their energy dissipation decreases from 4.12 fW to 2.15 fW attributed to cell morphological as well as migration characteristics variations.

(b) Computational perspective of flagellar dynamics. The above discussion related to the mechanical energy expenditure to maintain the cell motility. However, the source of the motility comes from the motion of the flagella. A look into the rate of work variation of the flagella across growth stage therefore strongly puts the above discussion into perspective. For the estimation of the rate of work in a flagella beating, we attempt an experimental procedure detailed in Figure S22. We extract the wavelength, frequency, and amplitude of the flagella from our experiments as shown previously. In this context, we discuss the deliberate reduction in mechanical work input from the cell flagella at late stationary stages. It has been observed that at stationary stage (nutrient depleted cultures), the fluid rheology tends to be more viscoelastic in nature (*75*, *76*). Such environmental changes have great ramifications in the context of flagella movement. This, in turn, relates to the focus of the study that a behavioral strategy emerges wherein a cell consciously attempts to save energy during the nutrient depleted environments. To proceed with the discussion, we assume Jeffrey fluid characteristics of the medium which is a linearized viscoelastic model having the constitutive mode that reads $(1+\lambda_1\partial/\partial t)\ \sigma = \eta\ (1+ \lambda_2\partial/\partial t)\ \dot{\gamma}$. We also assume that the properties of flagella do not drastically vary across the growth stage. With these considerations, we employ the result of the viscoelastic resistive force theory to compare the rate of work of the waving slender filament around different rheological environments. For a filament waving (beating) at single mode, the ratio of the rate of work in a Jeffrey fluid to the same in a Newtonian fluid is given as $\dot{W}_V/\dot{W}_N = (1+De_1De_2)/(1+De_1^2)$ (*74*) where De is the Deborah number defined as $\lambda\omega$. The subscripts *1* and *2* denote the two relaxation time scales in Jeffrey fluid. Since for Jeffrey fluid $\lambda_1>\lambda_2$, we have $De_1>De_2$ and thus the ratio $\dot{W}_V/\dot{W}_N<1$. In other words, a viscoelastic environment incurs a reduced rate of work for the same flagellar motion. However, linear viscoelastic fluids are known to violate the frame independence in their constitutive model and thus a nonlinear viscoelastic model reflects a realistic scenario.

For nonlinear viscoelasticity, a regular perturbation is used to obtain the ratio of the rate of work for viscoelastic and Newtonian fluids for a waving sheet (two-dimensional flow).

Considering Oldroyd-B as a model nonlinear viscoelastic model (*74*), we again observe that the ratio of viscoelastic to Newtonian rate of work is given by $\dot{W}_V/\dot{W}_N = (1+De_1De_2)/(1+De_1^2)$. Furthermore, the ratio of the swimming speeds for a flexible waving sheet in viscoelastic fluid to the same in a Newtonian fluid is given as $\dot{U}_V/\dot{U}_N = (1+De_1De_2)(1+4Sp^6)/(1+De_1^2+4Sp^6(1+De_2^2)+4Sp^3(De_1-De_2))$ (*74*) where $Sp=\eta\omega/Bk^3$ is known as the Sperm number. Here *B* is the bending rigidity ($<10^{-21}$ N/m^2,(*77*)), η is the medium viscosity and k is the wavenumber. Thus, for a highly flexible flagella, $Sp \ll 1$, the ratio becomes $\dot{U}_V/\dot{U}_N = (1+De_1De_2)/(1+De_2^2)$ (*74*). Since $De_1 > De_2$, the swimming velocity for a given flagellar motion is higher. In favor of approximate analytical results, such enhancement in swimming speeds and efficiencies are also reported in numerical simulations (*78*). Therefore, the overall message is clear; a cell needs to spend much less work to keep up the same swimming speed. In our experiments we observe that the swimming speed reduces nearly 50% from exponential stage to stationary stage. We also observe that the flagellar motion reduces when going into the stationary stage (Figure S22). Thus, a cell thereby consciously saves on mechanical flagellar energy rendering their environments viscoelastic. This, in turn, helps the cell to swim for longer times as a bet-hedging strategy in order to find new sources of nutrients.

ST3. Co-existence of strains under nutrient depleted environments. LD-based energy storage and strain-specific migratory shifts act concertedly as coupled traits to enhance fitness of nutrient-limited phytoplankton populations. Naturally co-existing strains of motile phototrophic *H. akashiwo*, S-1 and S-2, execute diel vertical migration under nutrient replete conditions. The phytoplankton population carry out photosynthesis, shuttling between light-rich photic zone during the day time and nutrient-rich depths at night. While S-1 exhibits stronger motility (high ballisticity and low reorientation time), and higher photosynthetic efficiency (higher F_v/F_m and ETR_{max} values) relative to S-2, it is less adapted under high light conditions indicated by the lower non-photochemical quenching (NPQ) parameter. As nutrient limitation sets off, both strains generate cytoplasmic LDs, while distinct behavioral and physiological shifts co-emerge, indicating that the LD-based energy storage and emergent migratory behavior adapt differentially. In nutrient limited settings, generation of intracellular LDs and their directional translocation drive suppression of vertical migration in S-1, while for S-2, significant enhancement is measured. Enhancement of the vertical motility in S-2 is facilitated by the concomitant morphological change, specifically due to the loss of the rotational symmetry as cells turn flatter in shape (platelets), thereby reducing the viscous losses during swimming. The morphological shift allows S-2 cells to conserve energy while maintaining motility in the upper light-rich surface waters, while for S-1 cells, the loss of ballisticity and orientational stability drive them toward diffusive regime in deeper waters. Alongside, the increased NPQ and reduced F_v/F_m values of S-1 conform with our observation their trophic switch from photo- to phagotrophic mode of resource acquisition. S-2, on the other hand, increases their ballisticity and motility, thus increasing the residence time in shallow light-rich waters. A relatively lower NPQ shows their enhanced ability to use the incident light toward photochemical processes. Finally, the reduction of the cell size in S-1, and an increase in the overall cell size of S-2 indicate that the two strains diversify their strategies for resource acquisition under limited settings. Under limited concentration of nutrients, small, low motile cells with high surface area to cell volume ratios will have competitive advantage (a strategy potentially adopted by S-1). In contrast, for stable cell nutrient quota, increased size is advantageous (*2*), which may allow S-2 to acquire just enough nutrients, e.g., upon chanced molecular encounters, for growth and division thus enhancing fitness during nutrient limited conditions (inset, Figure 4I). These differential strain-specific responses suggest that motile phytoplankton possess concerted decision-making mechanisms that allow contrasting adaptive strategies for acquiring a limited pool of nutrients, while maximization population-scale fitness. In doing so, phytoplankton redistribute along the vertical column, thus cumulatively covering larger physical space, while minimizing the overlap and potential inter-strain competion for limited resources.

Supplementary Figures

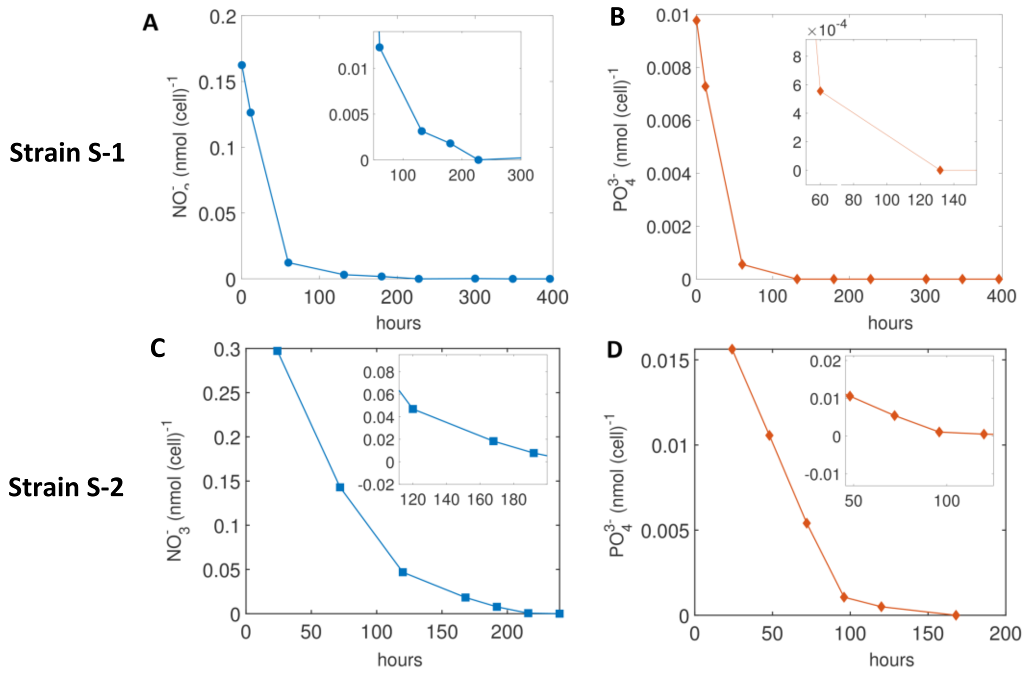

Fig. S2. Consumption of nitrate and phosphate nutrients in S-1 and S-2. (A) Time series of available nitrate NO_3^- [nmol] per cell for S-1 cultures grown in nutrient replete (C_0) f/2(-Si) medium, 22°C. Inset: zoomed-in view of the time series consumption of NO_3^- [[nmol] per cell] in batch experiment. Nitrate is completely depleted in batch culture after 300 hours of incubation. **(B)** Time-series of available nitrate PO_4^{3-} [nmol] per cell for S-1 cultures growing in nutrient replete (C_0) f/2(-Si) medium, 22°C. Inset: zoomed-in view of the time-series of PO_4^{3-} [nmol] per cell consumption in batch experiment. **(C)** Denotes the corresponding values of NO_3^- concentration for S-2 population; and **(D)** denotes the PO_4^{3-} concentration for S-2. The sharp reduction quantified for both $N0_3^-$ and PO_4^{3-} (nmol cell^{-1}) during first 60-70 hours is the consequence of exponential cell propagations. The complete depletion observed for nitrate in batch culture after 300 hours corresponds to late exponential growth phase and consequent entry into stationary phase.

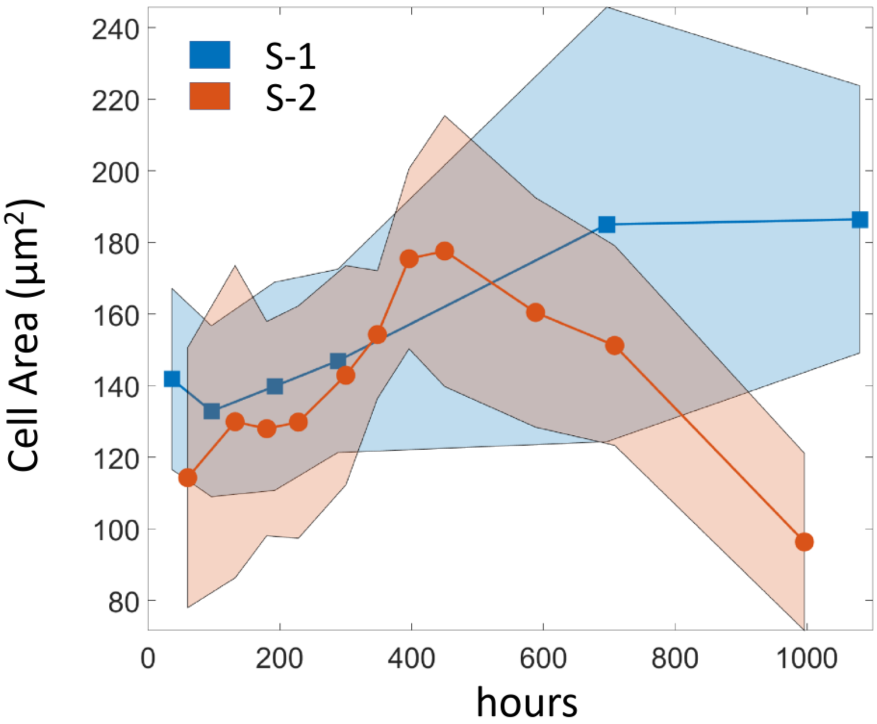

Fig. S3. Cell area during growth phase. Time series of the cell area as observed using bright-field microscopy imaging for strain S-1 (blue) and strain S-2 (orange). Comparison of S-1 and S-2 cell area variation in function of time in f/2 (-Si) replete nutrient concentration (C_0) at 22°C. Line and shaded area denote mean ± SD.

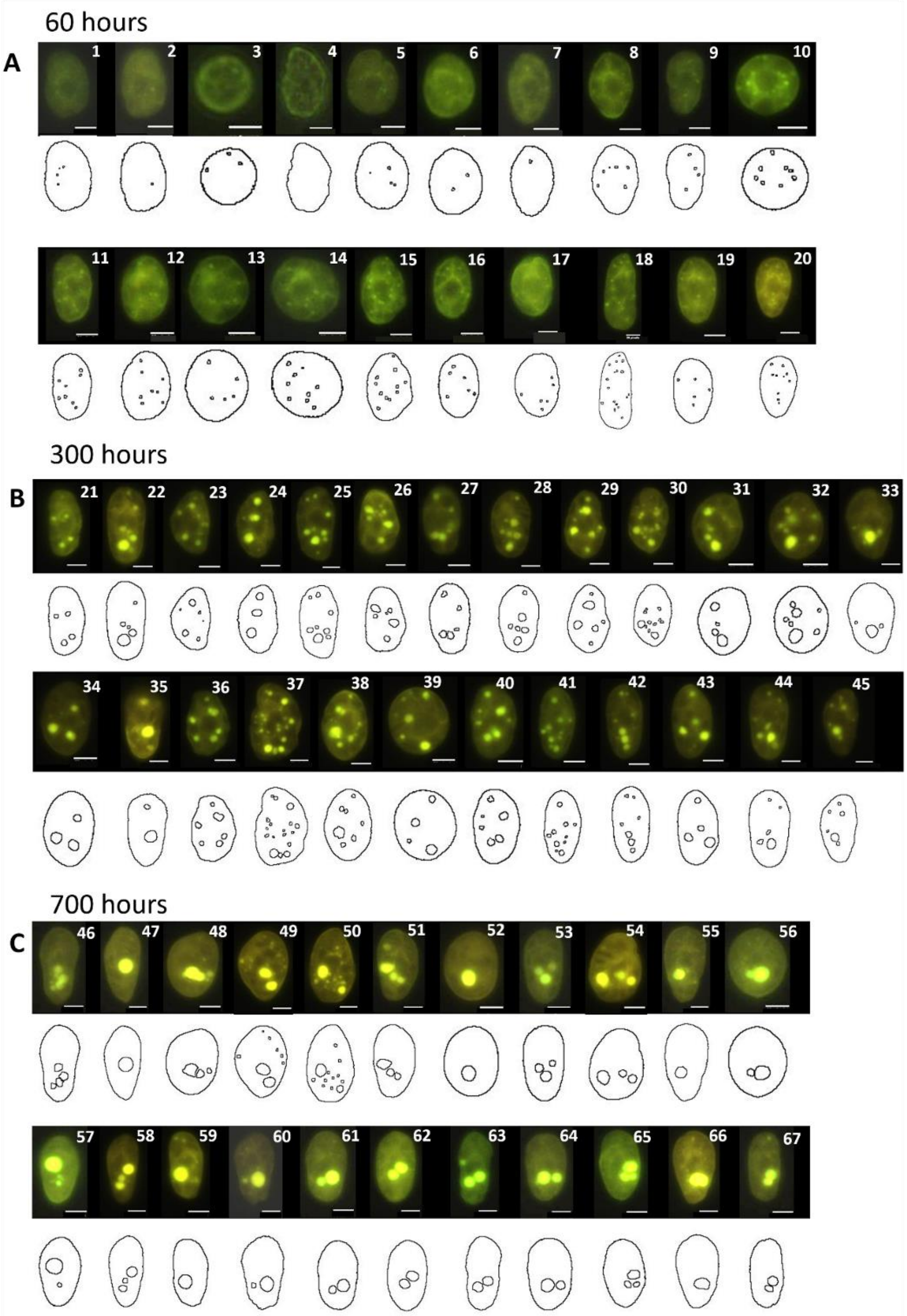

Fig. S4. Quantification of shape and lipid droplets position of S-1 cells based on single-cell microscopy, for time interval at 60 hours, 300 hours, 700 hours. (A-C) The top row in each panel shows micrographs obtained by epifluorescence microscopy (Methods), of S-1 cells collected at 60 hours (early exponential, n = 20) **(A)**, at 300 hours (late exponential, n = 24) **(B)** and 700 hours (stationary, n = 21) **(C)**. The cell itself was visualized using an inverted microscope (Olympus CKX53) in phase contrast, equipped with a 40x objective and an Imaging Source DFK33UX265 Camera. Prior to imaging, cells were stained with Nile Red (Methods) to visualize the intracellular neutral lipid droplets through fluorescence microscopy. Image analysis was used to extract the contour of each cell and the position of single lipid droplet in the cell (bottom row). Scale bar is 5 µm.

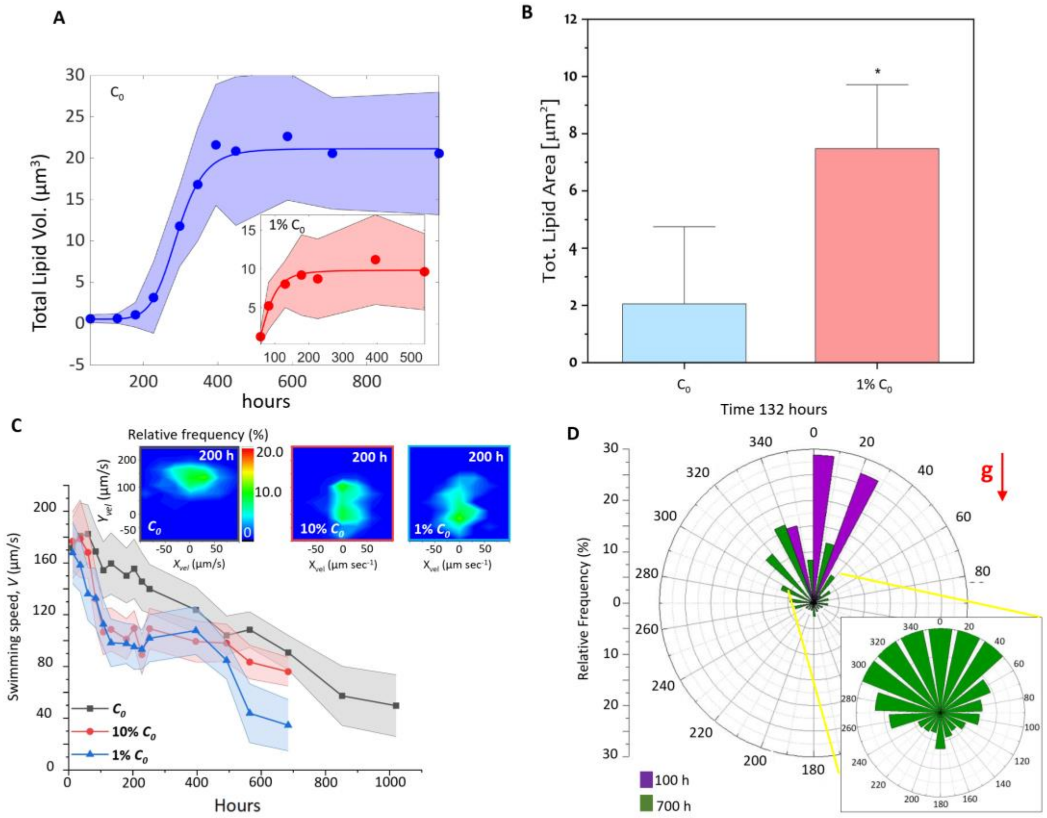

Fig. S5. Nutrient limitation accelerates lipid droplets accumulation in *H. akashiwo*. (A) Comparison of S-1 total lipid volume accumulation in function of time in f/2 (-Si) replete nutrient concentration (C_0) and in f/2 (-Si) with nutrients diluted 100 times (nutrient depleted, 1% C_0), 22°C. (**B**) At the same time (t = 132 hours), S-1 in nutrient replete (C_0) and nutrient deplete (1% C_0) show statistically significant difference in tot. lipid area. Mean ± 1SD; asterisks indicate statistical significance between the two samples (t-test for $p < 0.001$). **(C)** S-1 swimming speeds spanning the growth stages, for control (C_0, black), 10% C_0 (red), and 1% C_0 (blue) starting nutrient concentrations. For each starting concentration, the swimming speed peaked during early exponential stage, reducing thereafter. Inset (left to right): Joint velocity distributions (t = 200 h) for C_0, 10% C_0, and 1% C_0 reveal emergence of sub-populations with reduced vertical motility. **(D)** Nutrient-replete young S-1 population exhibits stronger swimming anisotropy along the vertical direction (against the gravity, **g,** t = 100 h, purple), relative to the older nutrient-limited population (t = 700 h, green). The zoomed-in view of the windrose center captures the angular distribution of the older population.

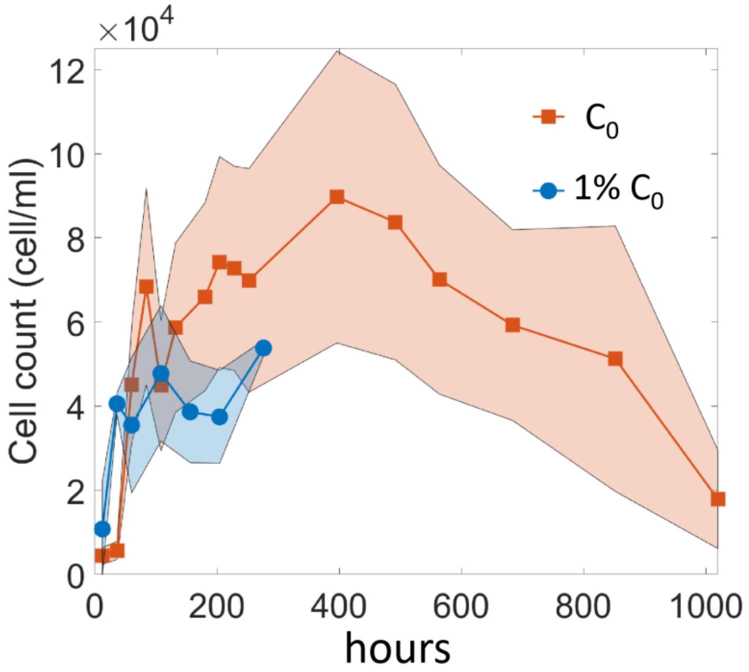

Fig. S6. Consequences of nutrient limitation on carrying capacity and motility. The temporal variation in cell concentration with different initial nutrient condition of C_0 and 1% C_0 where C_0 is the f/2(-Si) medium with initial $NaNO_3$ concentration being 0.882 mM and NaH_2PO_4 concentration being 0.0362 mM. Thus, carrying capacity at $C_0 \neq$ carrying capacity at 1% C_0. The corresponding cell count for S-2 may be found in Ref. (*36*).

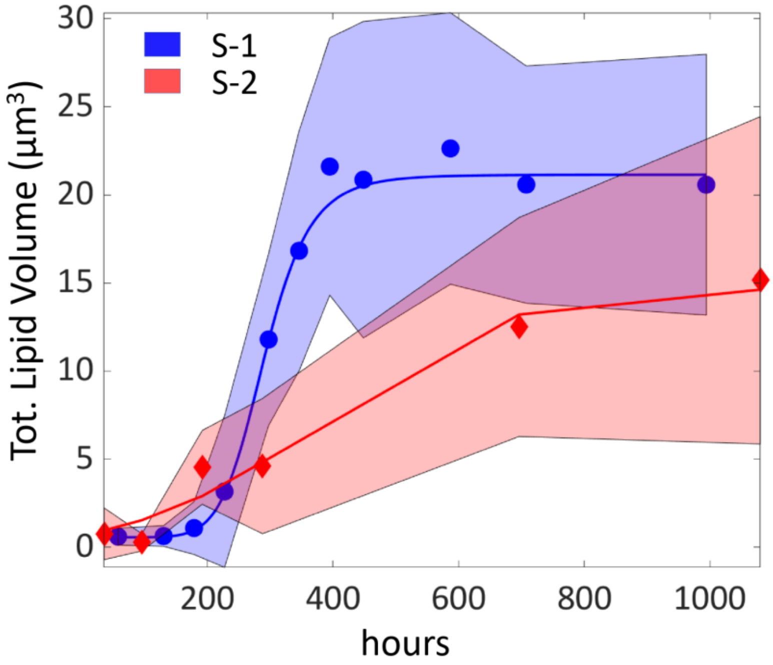

Fig. S7. Fitting: total lipid volume per cell does not change from 396 to 1000 hours. Total lipid volume quantified as the sum of each single LD volume in a cell (calculated using effective radius and assuming spherical shape of LDs). Number of analyzed S-1 and S-2 cells are more than 20 for each time point.

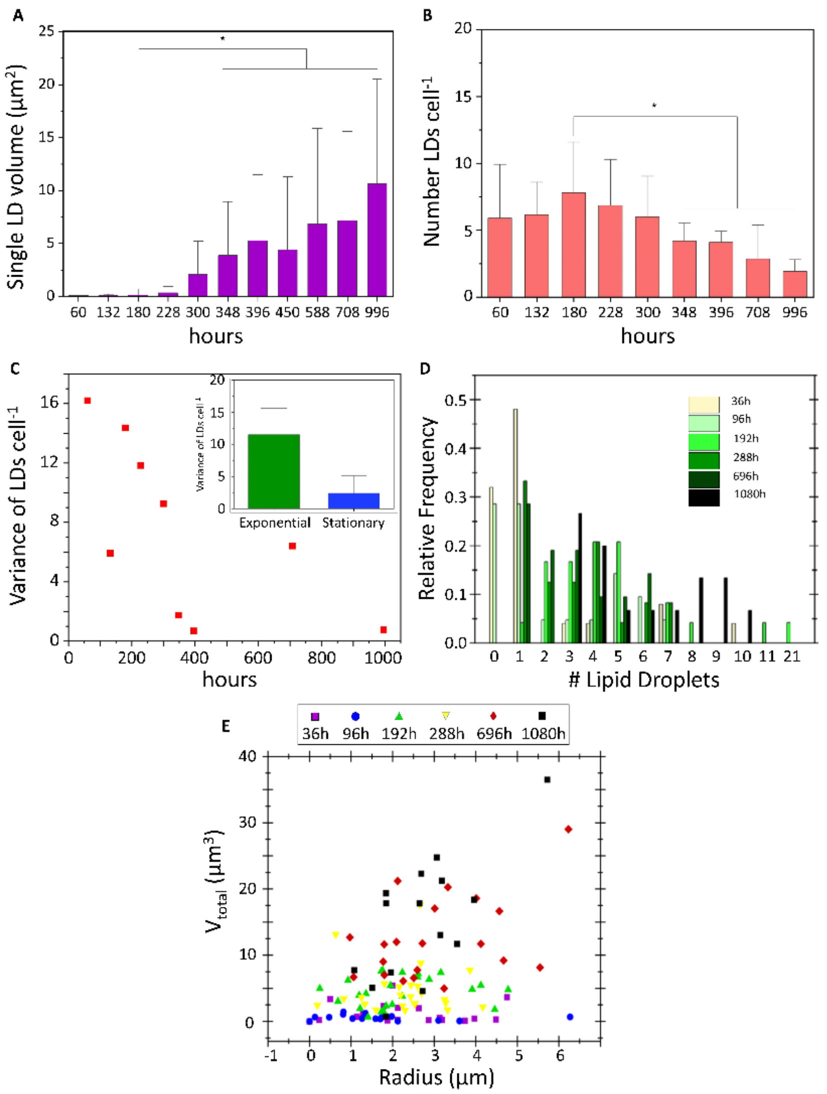

Fig. S8. Variation of LD volume and droplet distribution. (A) Time series quantification of single LD for S-1 volume [µm^3] shows opposite trend described for LD absolute number. Single LD volume increases over time and shows statistically significant difference between exponential and stationary phases. Bar represents mean ± s.d (asterisk show statistically significant difference, one-way ANOVA, $p < 0.001$, post-hoc Tukey's honest significant difference). The reduction in LDs absolute number and the increase in single LD volume point to an active coalescence phase-separation process that specifically mobilize LD organelle toward the bottom-half of S-1 cells. **(B)** Lipid droplets distribution for S-1: Number of analyzed S-1 cells: $n_{60} = 20$; $n_{132} = 20$; $n_{180} = 21$; $n_{228} = 21$; $n_{300} = 25$; $n_{348} = 30$; $n_{396} = 21$; $n_{708} = 22$; $n_{996} = 27$. Each dot represents variance at a given time point. Inset: bar plot mean variance ± s.d. at exponential and stationary phase. **(C)** Number of analyzed S-1 cells: $n_{60} = 20$; $n_{132} = 20$; $n_{180} = 21$; $n_{228} = 21$; $n_{300} = 25$; $n_{348} = 30$; $n_{396} = 21$; $n_{708} = 22$; $n_{996} = 27$. One Way ANOVA; significance level 0.001; Tukey's significant difference. **(D)** Relative frequency of number (#) of lipid droplets in one cell for different growth stages of S-2. **(E)** Total lipid volume V_{Total} in one cell in dependence of the distance r from the geometrical center of the cell to C_L for different growth stages. The number of analysed cells is n = 25 for 36 h, n = 21 for 96 h, n = 24 for 192 h, n = 24 for 288 h, n = 20 for 696 h and n = 15 for 1080 h.

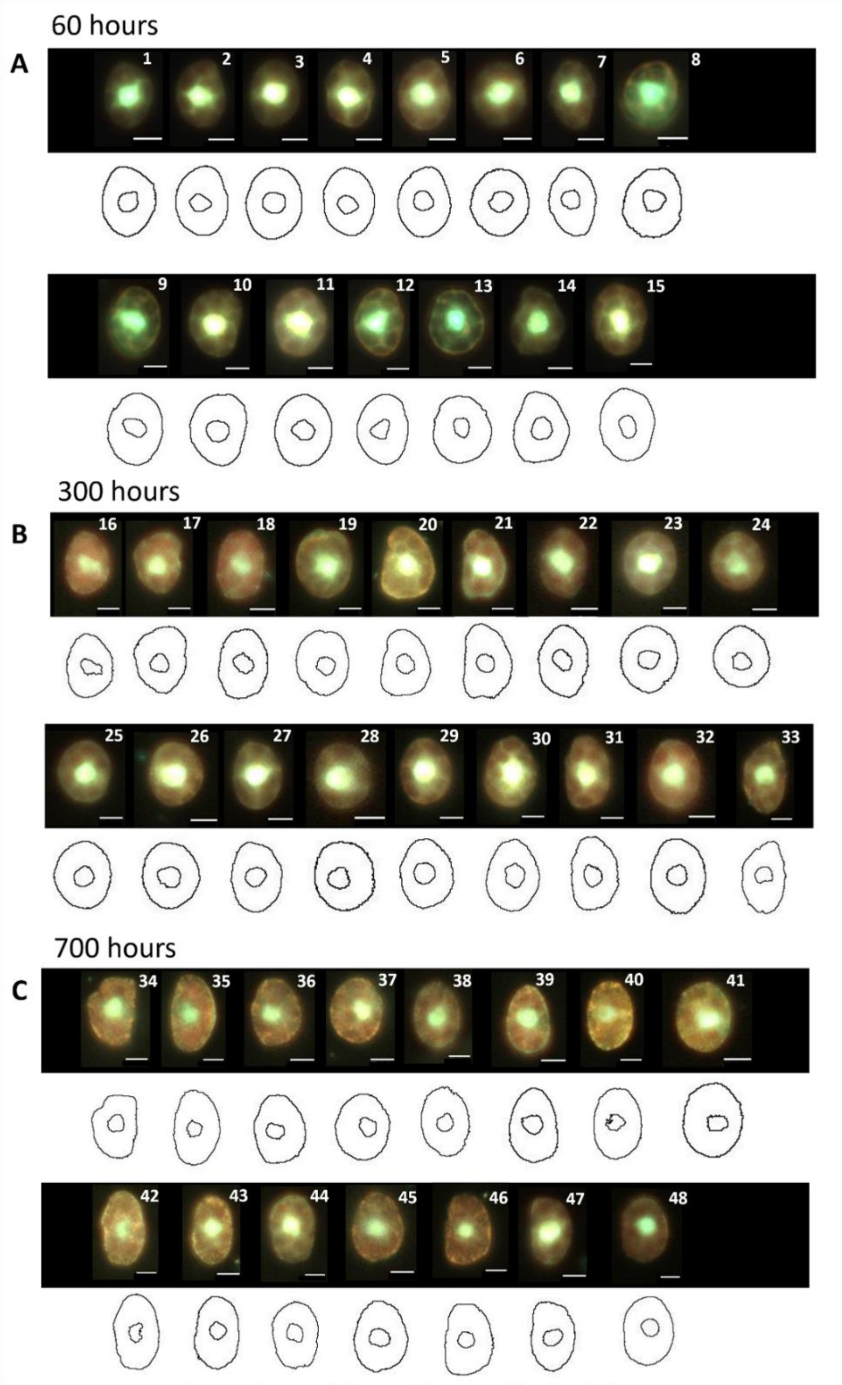

Fig.S9. Quantification of nucleus position of S-1 cells based on single-cell microscopy, for time interval at 60 hours, 300 hours, 700 hours. (A-C) The top row in each panel shows micrographs obtained by epifluorescence microscopy (Methods), of S-1 cells collected at 60 hours (early exponential, n = 15) (A), at 300 hours (late exponential, n = 18) (B) and 700 hours (stationary, n = 15) (C). The cell itself was visualized using an inverted microscope (Olympus CKX53) in phase contrast, equipped with a 40x objective and an Imaging Source DFK33UX265 Camera. Prior to imaging, cells were stained with Syto9 (Materials and Methods) to visualize the nucleus through fluorescence microscopy. Image analysis was used to extract the contour of each cell and the position of its nucleus. Scale bar is 5 µm. The nucleus radius for A, B, C is, respectively, 1.9 ± 0.18 µm, 1.9 ± 0.21 µm, 1.86 ± 0.15 µm. The distance from cell geometric center for A, B, C is, respectively, 0.40 ± 0.20 µm, 0.49 ± 0.21 µm, 0.56 ± 0.27 µm. Due to its proximity to cell geometric center the nucleus contribution to cell rotational stability is negligible compared to contribution of lipid droplets.

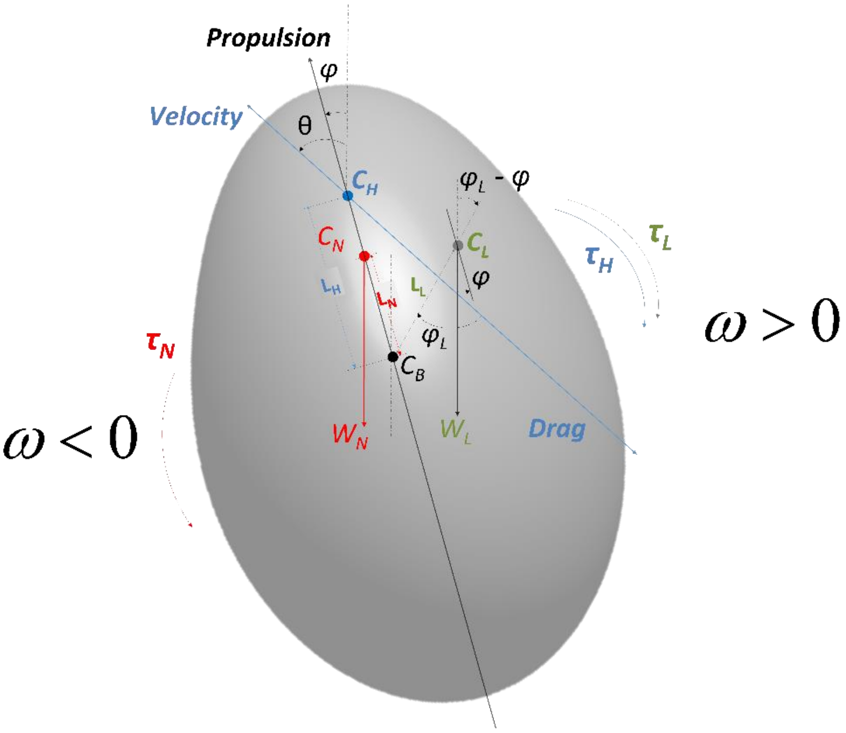

Fig. S10. Schematics of the geometry of the single cell of the phytoplankton along with the free body diagram of all forces and torques. Please refer to the Materials and Methods section (main text), and the Supplementary Text (ST1) for detailed equations.

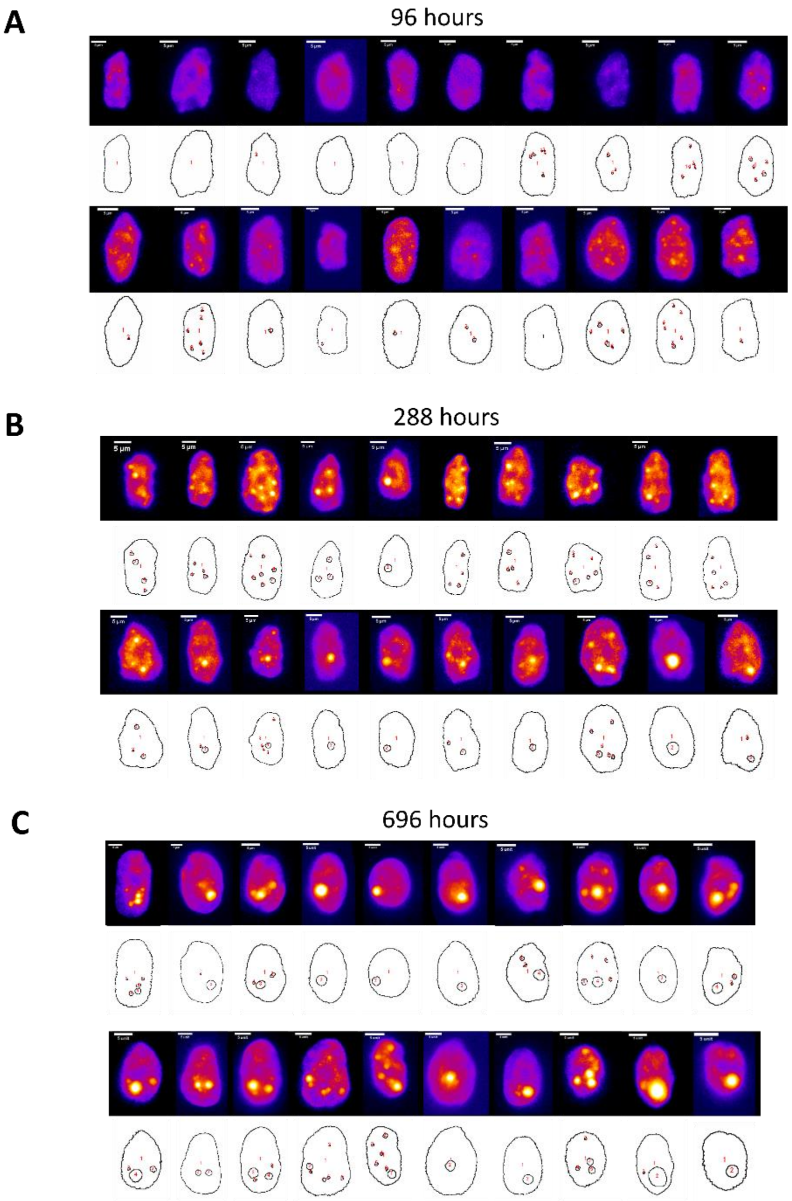

Fig. S11. Quantification of shape and lipid droplets position of S-2 cells based on single-cell microscopy, for time interval at 96 hours, 288 hours, 696 hours. (A-C) The top row in each panel shows micrographs obtained by epifluorescence microscopy (Materials and Methods), of S-2 cells collected at 96 hours (early exponential, n = 20) (A), at 288 hours (late exponential, n = 20) (B) and 696 hours (stationary, n = 20) (C). The cell itself was visualized using an inverted microscope (Olympus CKX53) in phase contrast, equipped with a 40x objective and an Imaging Source DFK33UX265 Camera. Prior to imaging, cells were stained with Nile Red (Methods) to visualize the intracellular neutral lipid droplets through fluorescence microscopy. Image analysis was used to extract the contour of each cell and the position of single lipid droplet in the cell (bottom row). Scale bar is 5 µm. The number of analysed cells is n = 25 for the time point t = 36 h, n = 21 for t = 96 h, n = 24 for t = 192 h, n = 24 for t = 288 h, n = 20 for t = 696 h and n = 15 for t = 1080 h. Error bars represent standard deviation over the analyzed samples.

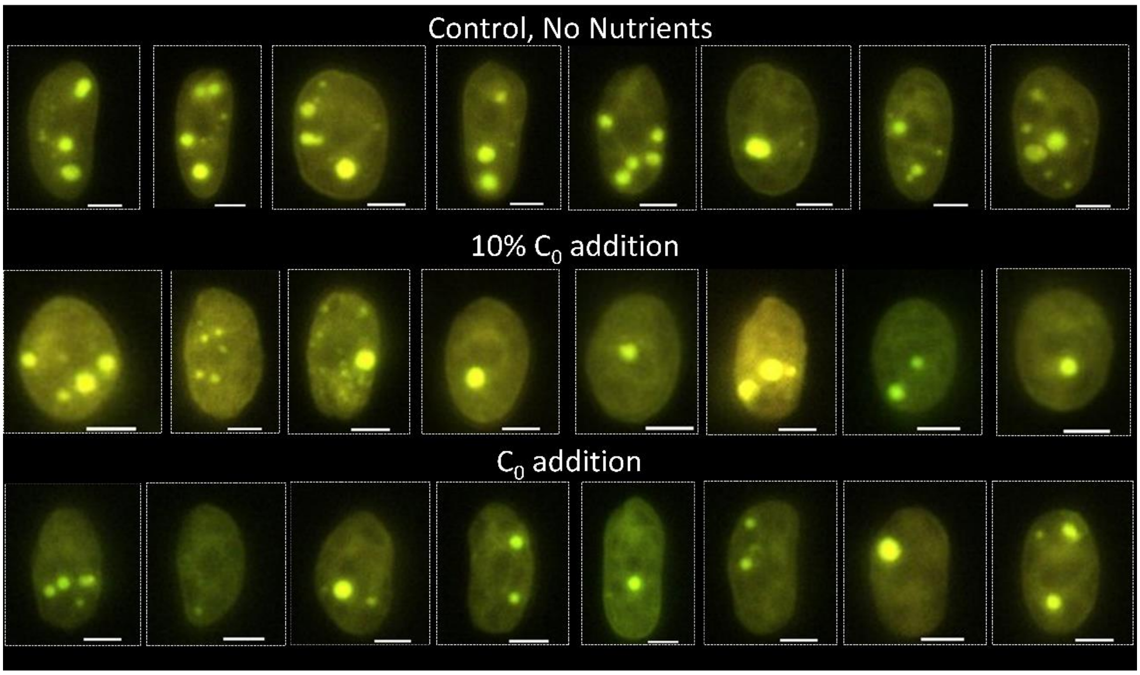

Fig. S12. Determination of lipid droplets in nutrient starved single cell S-1 24 hours after supplement of different concentration of fresh nutrients. Upon introduction of limited concentration of fresh nutrients, corresponding to 90x (10% C_0 addition) and 9x (C_0 addition) lower than optimal growth concentration), S-1 reduces its total intracellular lipid droplet volume. Corresponding to Figure S5, we observe that even during the recovery process, optimum concentration of nutrient re-supplementation drives recovery efficiently. Scale bar is 5 µm.

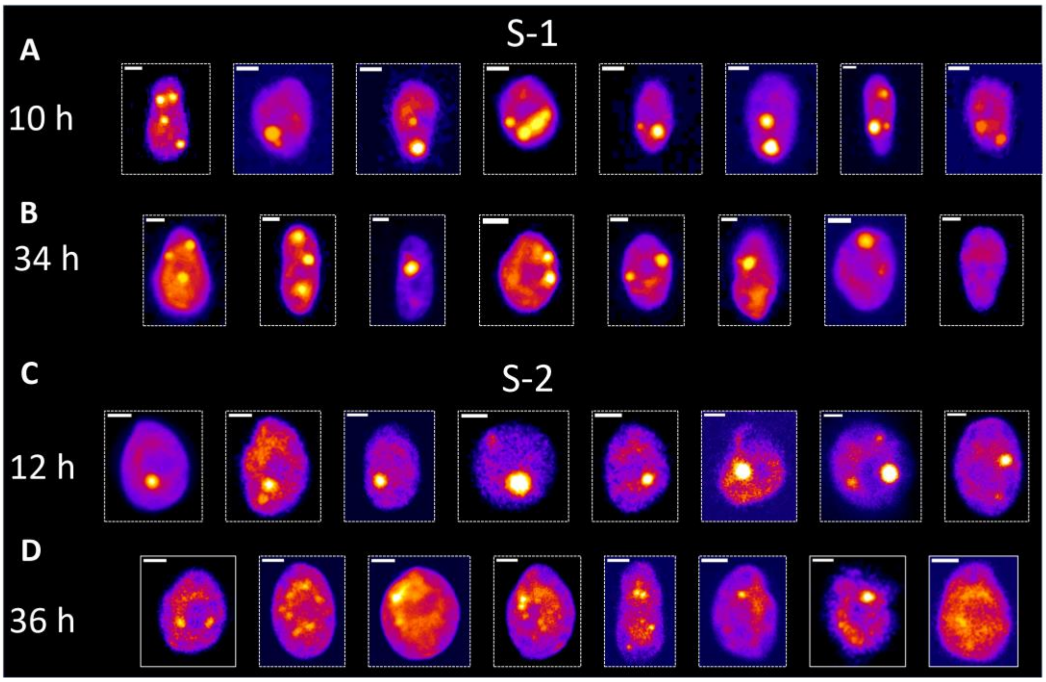

Fig. S13. Determination of lipid droplets in nutrient starved single cell S-1 and S-2 hours after supplement of fresh nutrients. Effect of introduction of fresh nutrients (Methods), the epifluorescence microscopy images of the lipid droplets distribution for **(A)** 10 h (S-1), **(B)** 34 h (S-1), **(C)** 12 h (S-2) and **(D)** 36 h (S-2). Upon introduction of limited concentration of fresh nutrients, both the strains reduce its total intracellular lipid droplet volume with time.

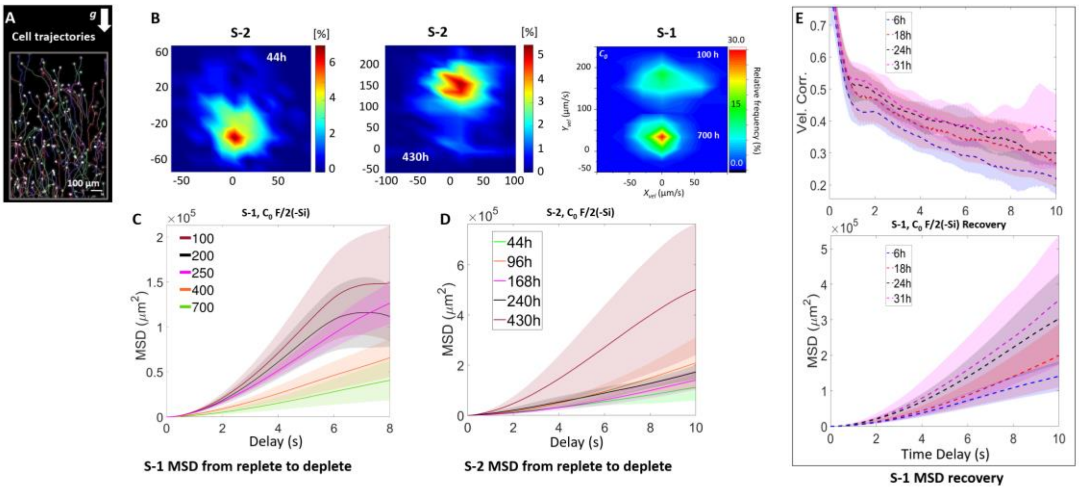

Fig. S14. Motility variation and its attributes across age for S-1 and S-2. (A) Trajectories of *H. akashiwo* cells in f/2(-Si) C_0 at 200 hours. **(B)** Joint velocity distribution at 44 h at C_0 (left), 430 h (middle) for S-2 and a comparison of 100 hours and 700 hours for S-1. Congruent with MSD (panel C) variation observed in panel D, the vertical velocity component (Y_{vel}) increases with nutrient-depletion. Nutrient depletion alters motility for both phenotype albeit in a contrasting manner. **(C)** MSD under nutrient replete condition (C_0) for S-1 at five time intervals representative for exponential (100 h, 200 h), late exponential (250 h) and stationary (400 h, 700 h) phases. MSD plot shows the variation in motility behavior occurring in phytoplankton population shifting from ballistic (during exponential) toward diffusive (during stationary) regimes. **(D)** MSD under nutrient replete condition (C_0) for S-2 at five time points representative for exponential (44, 96 and 168 h), late exponential (240 h) and stationary (430 h) phases. MSD plot shows the variation in motility behavior occurring in phytoplankton population shifting from ballistic (during exponential) toward diffusive (during stationary) regimes. **(E)** S-1 Recovery velocity correlation (top) mean square displacement and MSD (bottom) after re-supplementation of f/2(-Si) C_0 for 6h, 18h, 24h and 31h. Re-supplementation shows strong revival towards optimum velocity characteristics.

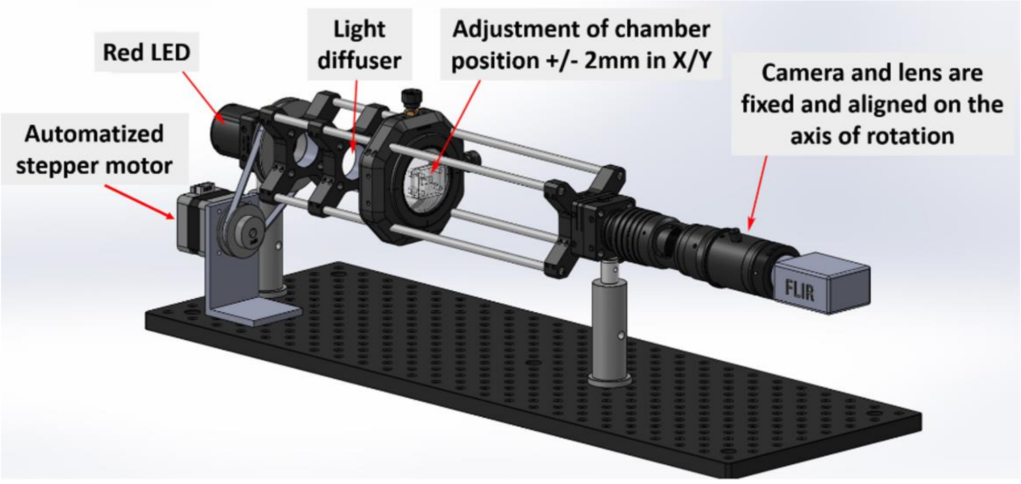

Fig. S15. Schematics of the microscopic imaging system that is used to find the reorientation time scale of the phytoplankton population.

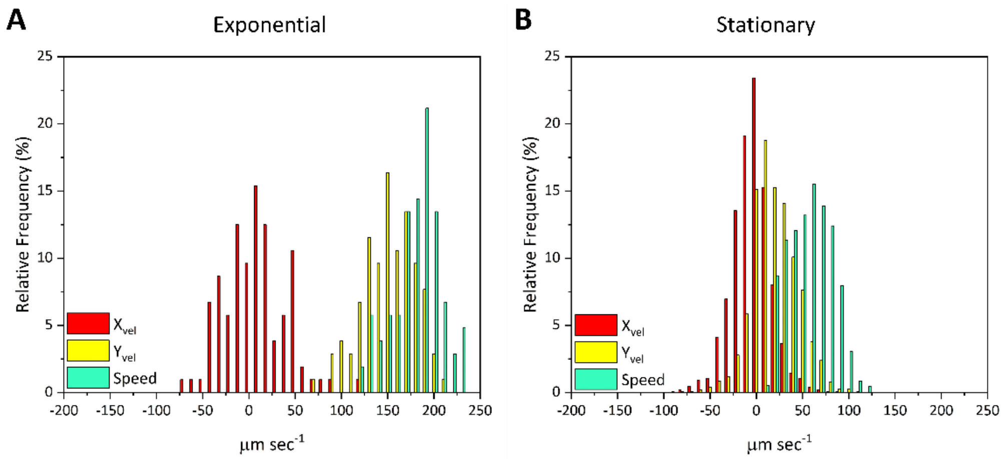

Fig. S16. Relative frequency of speed, vertical velocity and horizontal velocity components for S-1 at (A) exponential and (B) stationary phases. *H. akashiwo* 3107 grown in C_0 f/2(-Si), 22°C. At the exponential growth phase, the vertical velocity component (Y_{vel}) is the major contributor in phytoplankton speed. On the other side, the highest relative frequency for the horizontal velocity component (X_{vel}) to 0 µm sec^{-1} indicates no net displacement in the horizontal direction. Congruent with ballistic to diffusive motion transition between exponential to stationary growth phases presented in Figure 3, phytoplankton speed reduces at the stationary phase together with the reduction of vertical velocity component. In addition, a reduction in velocity anisotropy is also observed here. Anisotropy in velocity magnitude (ratio of y-directional velocity to x-directional velocity for upswimmers) of S-1 reduces with time. This supplements the velocity correlation plots delineating a reduction in the ballisticity. Overall, the population has a lower speed with diffusive motion. Using this distribution, we can deduce that the coefficient of anisotropy reduces, suggesting a shift from ballistic to diffusive migration.

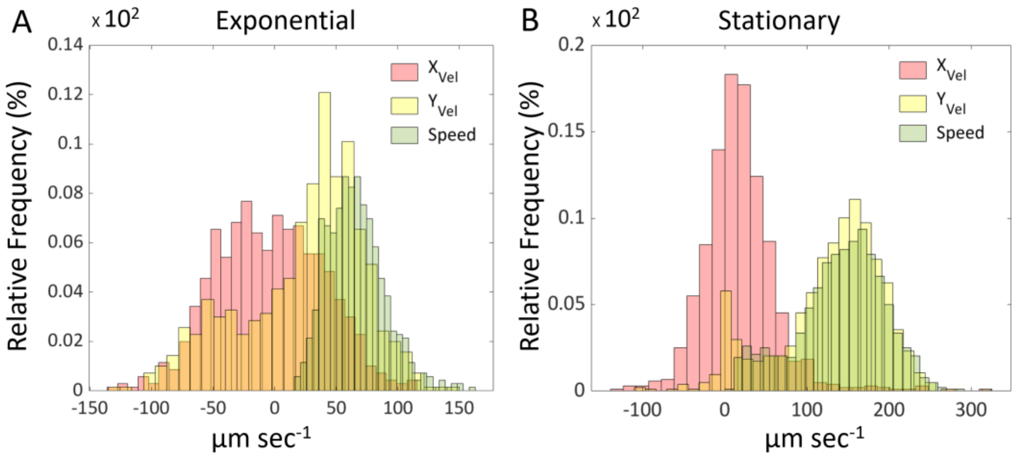

Fig. S17. Relative frequency of speed, vertical velocity and horizontal velocity components for S-2 at (A) exponential and (B) stationary phases. *H. akashiwo* 452 grown in C_0 f/2(-Si), 22°C. At the exponential growth phase, the vertical velocity component (Y_{vel}) is, in contrast to S-1, not the major contributor in phytoplankton speed. The highest relative frequency for the horizontal velocity component (X_{vel}) to 0 µm/sec indicates no net displacement in the horizontal direction. Congruent with diffusive to ballistic motion transition between exponential to stationary growth phases presented in Figure 3, phytoplankton speed increases at the stationary phase together with the increase of vertical velocity component. As a consequence, an increase in velocity anisotropy is also observed here, in contrast to S-1.

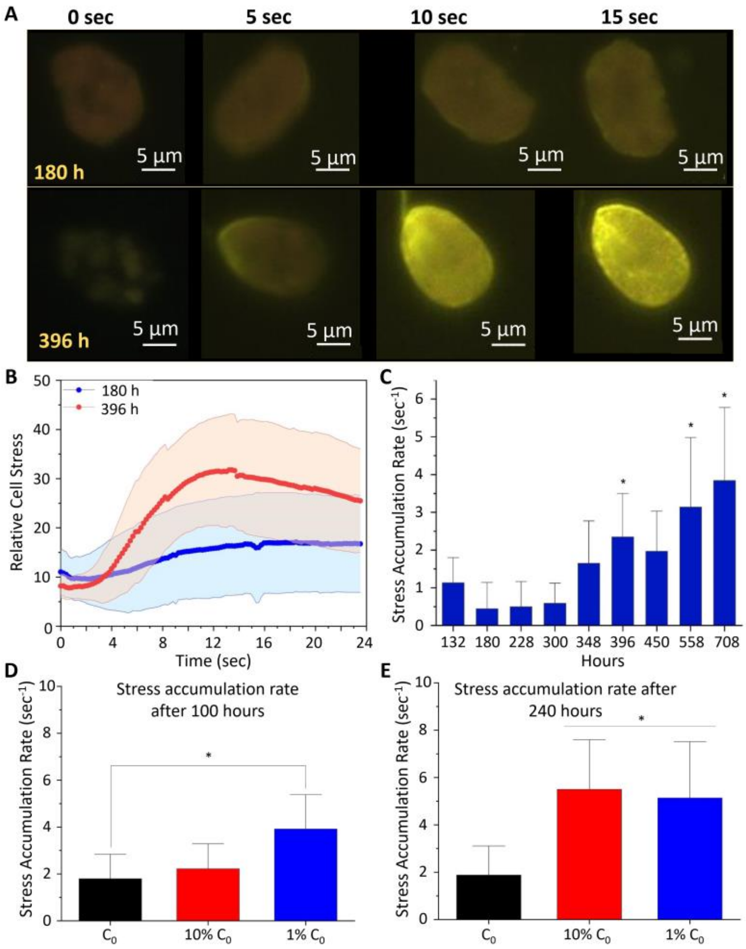

Fig. S18. Intracellular Reactive Oxygen Species (ROS) increases during the progressing growth phase. (A) Fluorescence intensity measuring production of ROS for S-1 at exponential (180 h) and early stationary (396 h). 5 µM CellROX Orange (Thermo Fisher), 30 min dark incubation. Excitation 545nm (green), 1/5 Exposure, 100% Intensity. **(B)** Time series of stress accumulation quantified as ROS production for cells after 180 h (blue) and after 396 h (red). Solid line represents arithmetic mean of analyzed cells and shaded regions represents mean ± s.d. Time represents the acquisition time under microscope. **(C)** From exponential to stationary: Rate of stress accumulation [sec^{-1}]. Asterisks at t = 396 h and t = 996 h indicate statistically significant difference relative to *t* =180 h (Mean ± SD. One Way ANOVA; significance level 0.001; Tukey's significant difference). Stress accumulation rate integrated over first 10 seconds of acquisition. Number of analyzed S-1 cells: $n_{132} = 20$; $n_{180} = 23$; $n_{228} = 23$; $n_{300} = 23$; $n_{348} = 16$; $n_{396} = 22$; $n_{708} = 25$; $n_{996} = 27$. **(D-E)** Stress accumulation rate (quantified as reactive oxygen species, ROS) enhances due to nutrient limitation. After 100 hours (D) stress accumulation rate for S-1 in 1% C_0 shows statistically significant difference to C_0; (E) after 240 hours both experiments under nutrient depleted conditions (10% C_0, 1% C_0) have higher stress accumulation rate than C_0. This result is congruent with the reduction in photosynthetic efficiency for 10% C_0 and 1% C_0 observed after 200 hours. (Bar represents mean ± s.d, and asterisks show statistically significant difference, one-way ANOVA, $p < 0.001$, post-hoc Tukey's honest significant difference).

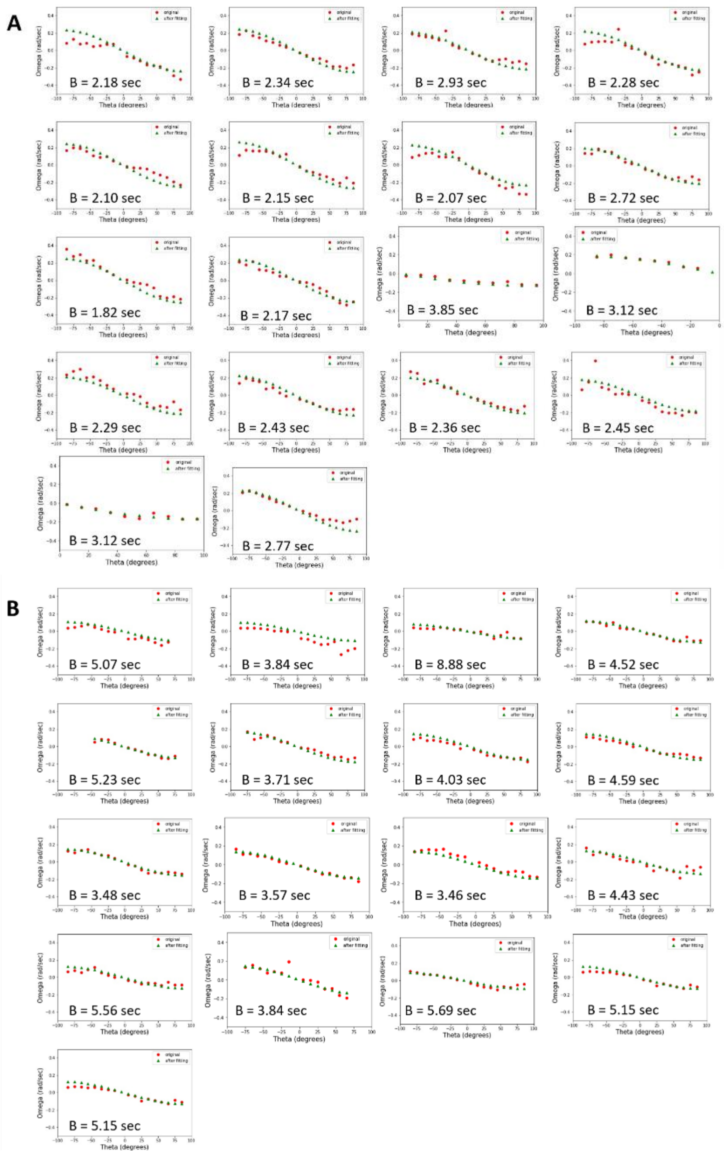

Fig. S19. Orientational stability of S-1 reduces in the stationary growth phase. Rotation rate (ω) as a function of the direction and angle (θ) of S-1 is shown for the exponential (72 hours, **(A)**) and the stationary (700 hours, **(B)**) growth phases. Plots show single replicate with experimental data (red) and the relative sinusoidal fit to the experimental data (green) used to obtain the reorientation timescale, *B*. The mean reorientation timescale obtained from these data are $B = 2.5 \pm 0.5$ s and $B = 4.7 \pm 1.3$ s for S-1 at, respectively, exponential, and stationary growth phases. The longer reorientation time occurring in phytoplankton at the stationary phase underpins the reduction in orientational stability due to intracellular LD accumulation and consequent generation of passive torque.

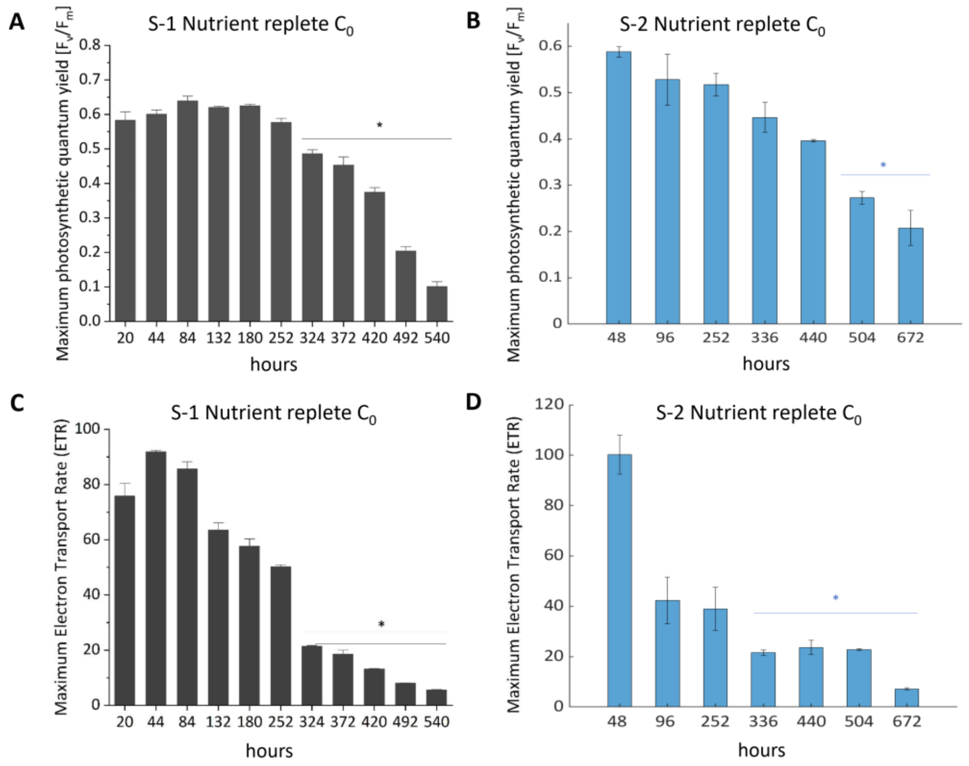

Fig. S20. Photosynthetic properties across growth phase for S-1 and S-2. (A-B) Statistically significant difference in F_v/F_m of (A) S-1 and (B) in f/2(-Si) C_0 is observed between exponential to stationary transition. Bar represents mean ± s.d (3 replicates); asterisks indicate statistically significant difference from F_v/F_m at the corresponding exponential growth stage (one-way ANOVA, $p < 0.001$, post-hoc Tukey's honest significant difference). Our experiments indicate that downregulation of S-1 photosynthetic efficiency is an active strategy that enable phytoplankton to save energy during LD-induced instability and emergent diffusive motion. **(C-D)** Time series of maximum electron transport rate of (C) S-1 and (D) S-2 in f/2(-Si) C_0. Bar represents mean ± s.d; asterisks indicate statistically significant difference from ETR_{max} at the corresponding exponential growth stage (one-way ANOVA, $p < 0.001$, post-hoc Tukey's honest significant difference). In general, both F_v/F_m and ETR_{max} decreases over the growth phase.

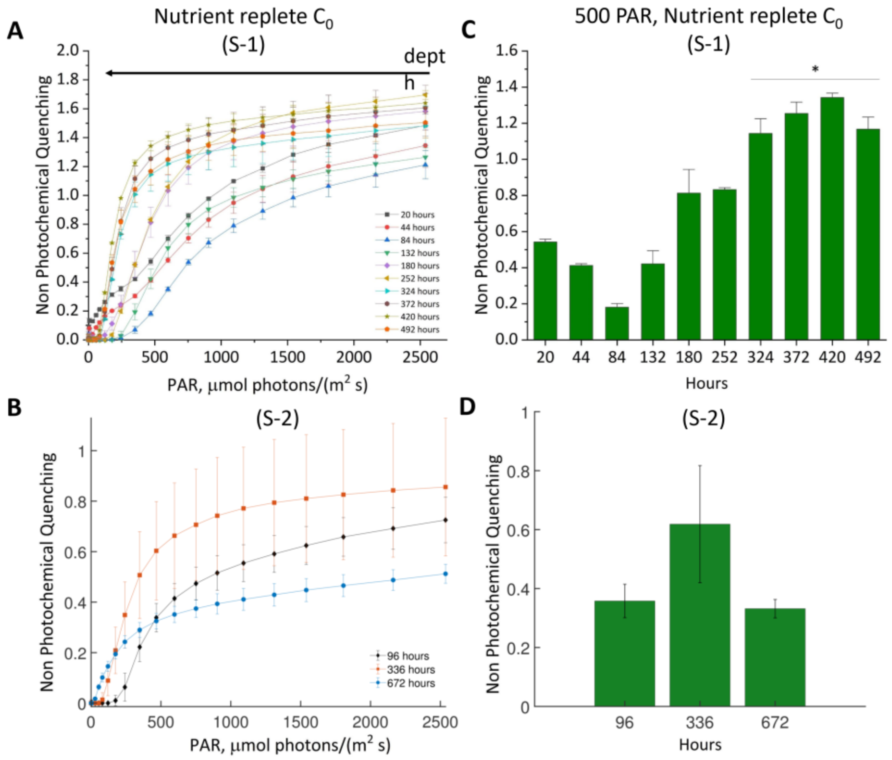

Fig. S21. Time series of non-photochemical quenching (NPQ) in response to gradual increase of photosynthetic active radiation (PAR, µmol photons/m^2 sec) in **(A)** S-1 and **(B)** S-2 under nutrient replete (C_0) conditions. Following the transition to stationary growth phase (> 324 hours) and reduction in photosynthetic efficiency described previously, S-1 and S-2 enhances NPQ at low PAR (< 500 µmol photons/m^2 sec), as a protective mechanism used to dissipate in the PSII system the excess of absorbed energy as heat. At 500 µmol photons/m^2 sec, **(C)** S-1 and **(D)** S-2 shows statistically significant difference in NPQ between exponential and stationary phase. This observation is congruent with the reduction in maximum photosynthetic quantum yield reported at the same time point, demonstrating that at stationary growth stage *H. akashiwo* reduces its photophysiological efficiency while inducing NPQ mechanism.

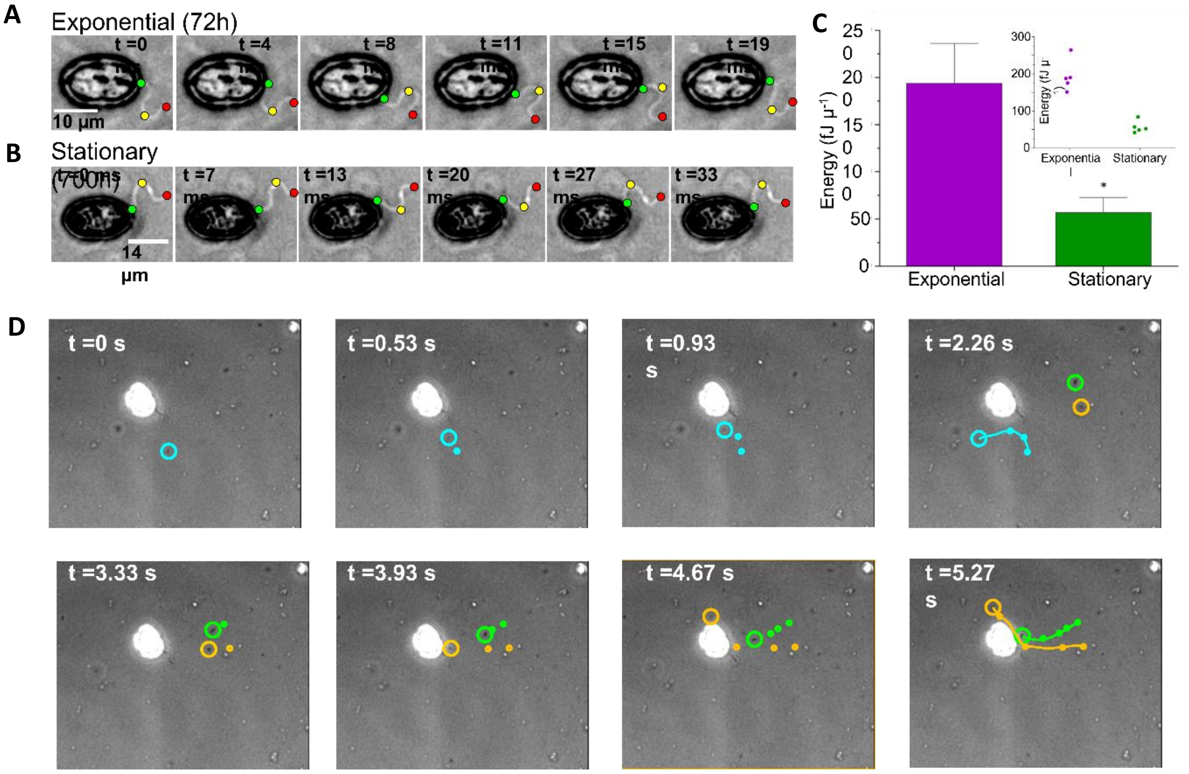

Fig. S22. Flagellar motion of S-1 and associated energy. S-1 flagellar beating at the exponential **(A)** and stationary phase **(B)**. 5 cells were chosen for each growth phase and their flagellar beating was studied under phase contrast microscope. To visualize cells under microscope, miniature 'swimming pools' were prepared as follows: On a glass slide, two (rectangular) pieces of scotch-tape were affixed about 1mm apart. A rectangular cover slip was placed on top of these and sealed on three sides. On the fourth side, cell culture was injected, and the samples was then observed under phase contrast mode (40x magnification). Videos were acquired at various frame rates (ranging between 200 to 350 fps), which were sufficiently high to cover successive flagellar strokes. **(C)** S-1 flagellar energy for the exponential and stationary phases are plotted using bar graphs showing the mean ± s.d (cells n=5), and asterisk indicates statistical difference between 72 hours (early exponential phase) and 700 hours (stationary phase). Two sample t-test, $p < 0.001$. Inset: energy scatter plot of single analyzed cell. **(D)** Trajectories of preys (beads and non-motile bacteria) reveal feeding currents generated by S-1 cells after prolonged nutrient limitation. No such feeding current was produced by nutrient - limited S-2 population.

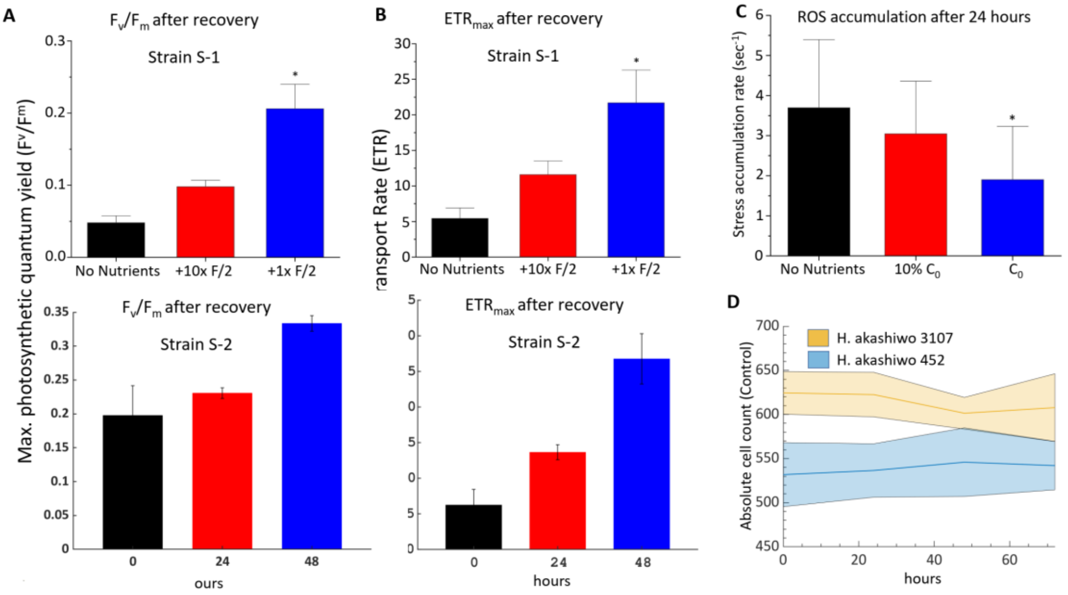

Fig. S23. Recovery of photosynthetic parameters and growth as an ecological strategy. (A) (TOP) Maximum photosynthetic quantum yield [F_v/F_m] after 24 hours of incubation. S-1 supplemented with C_0 f/2(-Si) shows statistically significant difference in F_v/F_m with both the control (no nutrients) and 10% C_0 f/2(-Si) (one-way ANOVA, p = 0.05, post-hoc Tukey's honest significant difference). After 24 hours, S-1 supplemented with C_0 f/2(-Si) has a 28% of recovery efficiency to optimal condition (F_v/F_m = 0.6, exponential phase). **(BOTTOM)** Maximum photosynthetic quantum yield [F_v/F_m] after 24 and 48 hours of incubation. S-2 supplemented with C_0 f/2(-Si) show strong increase with time. **(B) (TOP)** ETR_{max} rate after 24 hours of incubation. S-1 supplemented with C_0 f/2(-Si) shows statistically significant difference in ETR_{max} with both the control (no nutrient) and 10% C_0 f/2(-Si) (one-way ANOVA, p = 0.05, post-hoc Tukey's honest significant difference). **(BOTTOM)** ETR_{max} rate after 24 hours and 48 hours of incubation for S-2 cells again shows a strong recovery with time. **(C)** In accordance with the quantified increase in photophysiology, endogenous cellular stress rate (quantified as ROS) decreases after addition of fresh nutrients as compared to control populations which were not exposed to fresh nutrients after 24 hours of incubation (statistically significant difference was observed in presence of C_0 f/2(-Si) medium). Bar plots represent mean ± s.d (n = 13) and asterisk indicate statistically significant difference (one-way ANOVA, p = 0.05, post-hoc Tukey's honest significant difference). **(D)** Time series of the cell counts of the two strains at late stationary stage without addition of any nutrients (control conditions for 72 h cell count experiments).

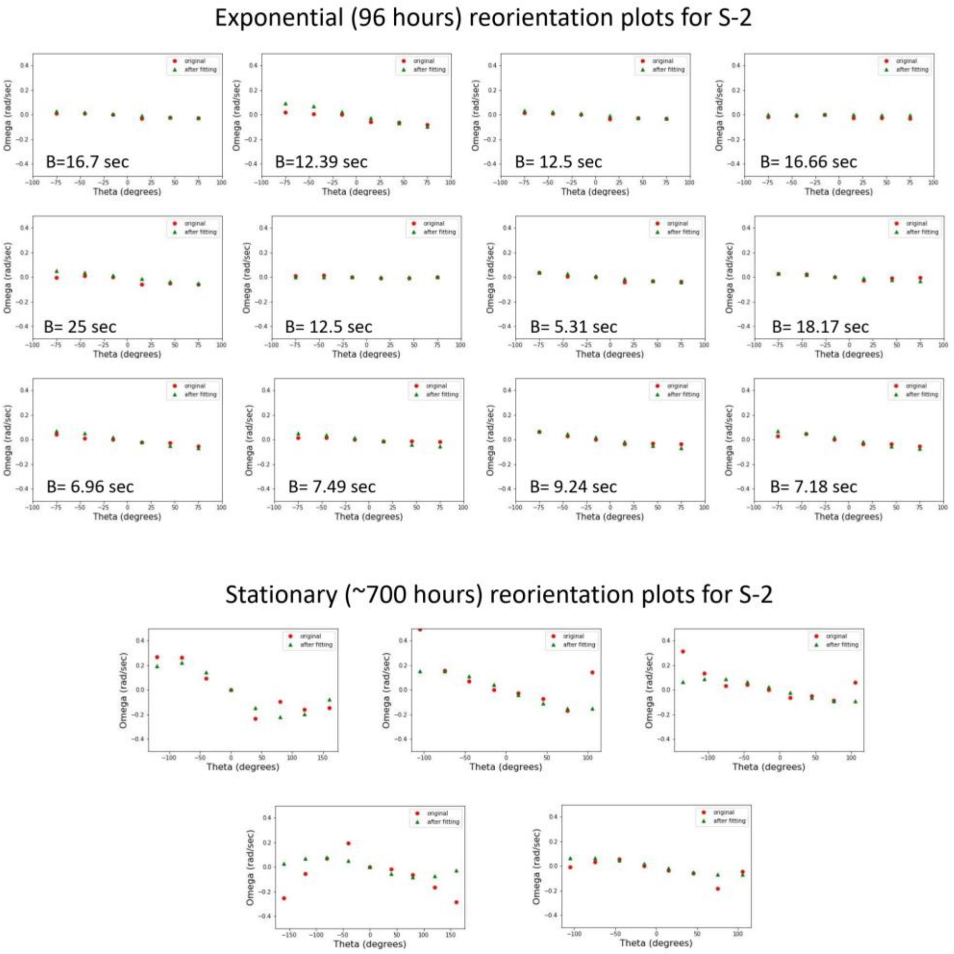

Fig. S24. Orientational stability of S-2 reduces in the stationary growth phase. Rotation rate (ω) as a function of the direction and angle (θ) of S-2 is shown for the exponential (96 hours, **(A)**) and the stationary (700 hours, **(B)**) growth phases. Plots show single replicate with experimental data (red) and the sinusoidal fit to the experimental data (green) used to obtain the reorientation timescale, *B*. The mean reorientation timescale obtained from these data are $B = 12.5 \pm 5$ s and $B = 5.11 \pm 2.16$ s for S-2 at exponential, and stationary growth phases respectively. The shorter reorientation time occurring in S-2 strain during the stationary phase reveals that an increase in swimming stability (negative gravitaxis) due to the variation in the cell morphology, specially, the loss of rotational symmetry of the cell shape (see Figure 4).

REFERENCES AND NOTES

1. D. B. Van de Waal, E. Litchman, Multiple global change stressor effects on phytoplankton nutrient acquisition in a future ocean. *Philos. Trans. R. Soc. B Biol. Sci.* **375**, 20190706 (2020).

2. C. M. Moore, M. M. Mills, K. R. Arrigo, I. Berman-Frank, L. Bopp, P. W. Boyd, E. D. Galbraith, R. J. Geider, C. Guieu, S. L. Jaccard, T. D. Jickells, J. La Roche, T. M. Lenton, N. M. Mahowald, E. Marañón, I. Marinov, J. K. Moore, T. Nakatsuka, A. Oschlies, M. A. Saito, T. F. Thingstad, A. Tsuda, O. Ulloa, Processes and patterns of oceanic nutrient limitation. *Nat. Geosci.* **6**, 701–710 (2013).

3. L. A. Bristow, W. Mohr, S. Ahmerkamp, M. M. M. Kuypers, Nutrients that limit growth in the ocean. *Curr. Biol.* **27**, R474–R478 (2017).

4. M. J. Behrenfeld, R. T. O'Malley, D. A. Siegel, C. R. McClain, J. L. Sarmiento, G. C. Feldman, A. J. Milligan, P. G. Falkowski, R. M. Letelier, E. S. Boss, Climate-driven trends in contemporary ocean productivity. *Nature* **444**, 752–755 (2006).

5. J. K. Moore, W. Fu, F. Primeau, G. L. Britten, K. Lindsay, M. Long, S. C. Doney, N. Mahowald, F. Hoffman, J. T. Randerson, Sustained climate warming drives declining marine biological productivity. *Science* **359**, 1139–1143 (2018).

6. J. J. Polovina, E. A. Howell, M. Abécassis, Ocean's least productive waters are expanding. *Geophys. Res. Lett.* **35**, L03618 (2008).

7. S. L. Hinder, G. C. Hays, M. Edwards, E. C. Roberts, A. W. Walne, M. B. Gravenor, Changes in marine dinoflagellate and diatom abundance under climate change. *Nat. Clim. Chang.* **2**, 271–275 (2012).

8. G. M. Hallegraeff, Ocean climate change, phytoplankton community responses, and harmful algal blooms: A formidable predictive challenge. *J. Phycol.* **46**, 220–235 (2010).

9. J. P. Mellard, K. Yoshiyama, E. Litchman, C. A. Klausmeier, The vertical distribution of phytoplankton in stratified water columns. *J. Theor. Biol.* **269**, 16–30 (2011).

10. K. Yoshiyama, J. P. Mellard, E. Litchman, C. A. Klausmeier, Phytoplankton competition for nutrients and light in a stratified water column. *Am. Nat.* **174**, 190–203 (2009).

11. C. A. Klausmeier, E. Litchman, Algal games: The vertical distribution of phytoplankton in poorly mixed water columns. *Limnol. Oceanogr.* **46**, 1998–2007 (2001).

12. M. Winder, U. Sommer, Phytoplankton response to a changing climate. *Hydrobiologia* **698**, 5–16 (2012).

13. E. D. Tobin, D. Grünbaum, J. Patterson, R. A. Cattolico, Behavioral and physiological changes during benthic-pelagic transition in the harmful alga, Heterosigma akashiwo: Potential for rapid bloom formation. *PLOS ONE* **8**, e76663 (2013).

14. M. B. Peacock, R. M. Kudela, Evidence for active vertical migration by two dinoflagellates experiencing iron, nitrogen, and phosphorus limitation. *Limnol. Oceanogr.* **59**, 660–673 (2014).

15. K. Richardson, J. Bendtsen, Vertical distribution of phytoplankton and primary production in relation to nutricline depth in the open ocean. *Mar. Ecol. Prog. Ser.* **620**, 33–46 (2019).

16. S. T. Haley, H. Alexander, A. R. Juhl, S. T. Dyhrman, Transcriptional response of the harmful raphidophyte Heterosigma akashiwo to nitrate and phosphate stress. *Harmful Algae* **68**, 258–270 (2017).

17. D. M. Anderson, J. M. Burkholder, W. P. Cochlan, P. M. Glibert, C. J. Gobler, C. A. Heil, R. M. Kudela, M. L. Parsons, J. E. J. Rensel, D. W. Townsend, V. L. Trainer, G. A. Vargo, Harmful algal blooms and eutrophication: Examining linkages from selected coastal regions of the United States. *Harmful Algae* **8**, 39–53 (2008).

18. P. Glibert, D. Anderson, P. Gentien, E. Granéli, K. Sellner, The global, complex phenomena of harmful algal blooms. *Oceanography* **18**, 136–147 (2005).

19. E. Litchman, C. A. Klausmeier, K. Yoshiyama, Contrasting size evolution in marine and freshwater diatoms. *Proc. Natl. Acad. Sci. U.S.A.* **106**, 2665–2670 (2009).

20. A. Beckmann, I. Hense, Torn between extremes: The ups and downs of phytoplankton. *Ocean Dyn.* **54**, 581–592 (2004).

21. O. Ross, J. Sharples, Phytoplankton motility and the competition for nutrients in the thermocline. *Mar. Ecol. Prog. Ser.* **347**, 21–38 (2007).

22. E. Litchman, C. A. Klausmeier, Trait-based community ecology of phytoplankton. *Annu. Rev. Ecol. Evol. Syst.* **39**, 615–639 (2008).

23. Z. V. Finkel, J. Beardall, K. J. Flynn, A. Quigg, T. A. V. Rees, J. A. Raven, Phytoplankton in a changing world: Cell size and elemental stoichiometry. *J. Plankton Res.* **32**, 119–137 (2010).

24. C. Lindemann, Ø. Fiksen, K. H. Andersen, D. L. Aksnes, Scaling laws in phytoplankton nutrient uptake affinity. *Front. Mar. Sci.* **3,** 1–6 (2016).

25. E. D. Galbraith, A. C. Martiny, A simple nutrient-dependence mechanism for predicting the stoichiometry of marine ecosystems. *Proc. Natl. Acad. Sci. U.S.A.* **112**, 8199–8204 (2015).

26. S. Zhu, W. Huang, J. Xu, Z. Wang, J. Xu, Z. Yuan, Metabolic changes of starch and lipid triggered by nitrogen starvation in the microalga Chlorella zofingiensis. *Bioresour. Technol.* **152**, 292–298 (2014).

27. L. Recht, A. Zarka, S. Boussiba, Patterns of carbohydrate and fatty acid changes under nitrogen starvation in the microalgae Haematococcus pluvialis and Nannochloropsis sp. *Appl. Microbiol. Biotechnol.* **94**, 1495–1503 (2012).

28. J. Msanne, D. Xu, A. R. Konda, J. A. Casas-Mollano, T. Awada, E. B. Cahoon, H. Cerutti, Metabolic and gene expression changes triggered by nitrogen deprivation in the photoautotrophically grown microalgae Chlamydomonas reinhardtii and Coccomyxa sp. C-169. *Phytochemistry* **75**, 50–59 (2012).

29. K. A. Fredrickson, S. L. Strom, R. Crim, K. J. Coyne, Interstrain variability in physiology and genetics of Heterosigma akashiwo (Raphidophyceae) from the West Coast Of North America1. *J. Phycol.* **47**, 25–35 (2011).

30. E. L. Harvey, S. Menden-Deuer, T. A. Rynearson, Persistent intra-specific variation in genetic and behavioral traits in the raphidophyte, Heterosigma akashiwo. *Front. Microbiol.* **6**, 1277 (2015).

31. H. J. Jeong, K. A. Seong, N. S. Kang, Y. Du Yoo, S. W. Nam, J. Y. Park, W. Shin, P. M. Glibert, D. Johns, Feeding by raphidophytes on the cyanobacterium synechococcus sp. *Aquat. Microb. Ecol.* **58**, 181–195 (2010).

32. H. J. Jeong, Mixotrophy in red tide algae raphidophytes1. *J. Eukaryot. Microbiol.* **58**, 215–222 (2011).

33. R. Martinez, E. Orive, A. Laza-Martinez, S. Seoane, Growth response of six strains of Heterosigma akashiwo to varying temperature, salinity and irradiance conditions. *J. Plankton Res.* **32**, 529–538 (2010).

34. R. Schuech, S. Menden-Deuer, Going ballistic in the plankton: Anisotropic swimming behavior of marine protists. *Limnol. Oceanogr. Fluids Environ.* **4**, 1–16 (2014).

35. H. Kim, A. J. Spivack, S. Menden-Deuer, pH alters the swimming behaviors of the raphidophyte Heterosigma akashiwo: Implications for bloom formation in an acidified ocean. *Harmful Algae* **26**, 1–11 (2013).

36. A. Sengupta, F. Carrara, R. Stocker, Phytoplankton can actively diversify their migration strategy in response to turbulent cues. *Nature* **543**, 555–558 (2017).

37. A. Engesmo, W. Eikrem, S. Seoane, K. Smith, B. Edvardsen, A. Hofgaard, C. R. Tomas, New insights into the morphology and phylogeny of Heterosigma akashiwo (Raphidophyceae), with the description of Heterosigma minor sp. nov. *Phycologia* **55**, 279–294 (2016).

38. R. Margalef, Life-forms of phytoplankton as survival alternatives in an unstable environment. *Oceanol. Acta* **1**, 493–509 (1978).

39. J. P. Grover, Is storage an adaptation to spatial variation in resource availability? *Am. Nat.* **173**, E44–E61 (2009).

40. J. P. Grover, Sink or swim? Vertical movement and nutrient storage in phytoplankton. *J. Theor. Biol.* **432**, 38–48 (2017).

41. S. Malitsky, C. Ziv, S. Rosenwasser, S. Zheng, D. Schatz, Z. Porat, S. Ben-Dor, A. Aharoni, A. Vardi, Viral infection of the marine alga Emiliania huxleyi triggers lipidome remodeling and induces the production of highly saturated triacylglycerol. *New Phytol.* **210**, 88–96 (2016).

42. J. A. Olzmann, P. Carvalho, Dynamics and functions of lipid droplets. *Nat. Rev. Mol. Cell Biol.* **20**, 137–155 (2019).

43. N. Guéguen, D. Le Moigne, A. Amato, J. Salvaing, E. Maréchal, Lipid droplets in unicellular photosynthetic stramenopiles. *Front. Plant Sci.* **12**, 639276 (2021).

44. A. R. Thiam, R. V. Farese Jr., T. C. Walther, The biophysics and cell biology of lipid droplets. *Nat. Rev. Mol. Cell Biol.* **14**, 775–786 (2013).

45. J. P. Grover, Resource competition in a variable environment: Phytoplankton growing according to the variable-internal-stores model. *Am. Nat.* **138**, 811–835 (1991).

46. K. F. Edwards, M. K. Thomas, C. A. Klausmeier, E. Litchman, Allometric scaling and taxonomic variation in nutrient utilization traits and maximum growth rate of phytoplankton. *Limnol. Oceanogr.* **57**, 554–566 (2012).

47. N. Ji, L. Lin, L. Li, L. Yu, Y. Zhang, H. Luo, M. Li, X. Shi, D.-Z. Wang, S. Lin, Metatranscriptome analysis reveals environmental and diel regulation of a Heterosigma akashiwo (raphidophyceae) bloom. *Environ. Microbiol.* **20**, 1078–1094 (2018).

48. N. Ji, Z. Zhang, J. Huang, L. Zhou, S. Deng, X. Shen, S. Lin, Utilization of various forms of nitrogen and expression regulation of transporters in the harmful alga Heterosigma akashiwo (Raphidophyceae). *Harmful Algae* **92**, 101770 (2020).

49. T. Smayda, Ecophysiology and bloom dynamics of Heterosigma akashiwo (Raphidophyceae). *Physiol. Ecol. Harmful Algal Bloom.* (1998).

50. T. J. Hansen, M. Hondzo, M. T. Mashek, D. G. Mashek, P. A. Lefebvre, Algal swimming velocities signal fatty acid accumulation. *Biotechnol. Bioeng.* **110**, 143–152 (2013).

51. J. You, K. Mallery, D. G. Mashek, M. Sanders, J. Hong, M. Hondzo, Microalgal swimming signatures and neutral lipids production across growth phases. *Biotechnol. Bioeng.* **117**, 970–980 (2020).

52. S. D. Rokitta, P. von Dassow, B. Rost, U. John, P- and N-depletion trigger similar cellular responses to promote senescence in eukaryotic phytoplankton. *Front. Mar. Sci.* **3**, 1–13 (2016).

53. F. Carrara, A. Sengupta, L. Behrendt, A. Vardi, R. Stocker, Bistability in oxidative stress response determines the migration behavior of phytoplankton in turbulence. *Proc. Natl. Acad. Sci. U.S.A.* **118**, e2005944118 (2021).

54. C. McLean, S. T. Haley, G. J. Swarr, M. C. K. Soule, S. T. Dyrhman, E. B. Kujawinski, Harmful algal bloom-forming organism responds to nutrient stress distinctly from model phytoplankton. bioRxiv 2021.02.08.430350 [Preprint]. 10 February 2021. https://doi.org/10.1101/2021.02.08.430350.

55. S. Hatano, Y. Hara, M. Takahashi, Photoperiod and nutrients on the vertical migratory behavior of a red tide flagellate, Heterosigma akashiwo. *J. Jpn. Phycol.* **31**, 263–269 (1983).

56. M. Wada, Y. Hara, M. Kato, M. Yamada, T. Fujii, Diurnal appearance, fine structure, and chemical composition of fatty particles in Heterosigma akashiwo (Raphidophyceae). *Protoplasma* **137**, 134–139 (1987).

57. N. R. Baker, Chlorophyll fluorescence: A probe of photosynthesis in vivo. *Annu. Rev. Plant Biol.* **59**, 89–113 (2008).

58. J. J. Cullen, J. G. MacIntyre, Behavior, physiology and the niche of depth-regulating phytoplankton. *Nato Asi Ser. G Ecol. Sci.* **41**, 559–580 (1998).

59. M. B. Lakeman, P. von Dassow, R. A. Cattolico, The strain concept in phytoplankton ecology. *Harmful Algae* **8**, 746–758 (2009).

60. A. K. Yamazaki, D. Kamykowski, A dinoflagellate adaptive behavior model: Response to internal biochemical cues. *Ecol. Model.* **134**, 59–72 (2000).

61. S. Menden-Deuer, J. Rowlett, M. Nursultanov, S. Collins, T. Rynearson, Biodiversity of marine microbes is safeguarded by phenotypic heterogeneity in ecological traits. *PLOS ONE* **16**, e0254799 (2021).

62. K. J. Flynn, Ecological modelling in a sea of variable stoichiometry: Dysfunctionality and the legacy of redfield and monod. *Prog. Oceanogr.* **84**, 52–65 (2010).

63. G. E. Hutchinson, The paradox of the plankton. *Am. Nat.* **95**, 137–145 (1961).

64. L. Li, P. Chesson, The effects of dynamical rates on species coexistence in a variable environment: The paradox of the plankton revisited. *Am. Nat.* **188**, E46–E58 (2016).

65. N. Tarantino, J.-Y. Tinevez, E. F. Crowell, B. Boisson, R. Henriques, M. Mhlanga, F. Agou, A. Israël, E. Laplantine, TNF and IL-1 exhibit distinct ubiquitin requirements for inducing NEMO–IKK supramolecular structures. *J. Cell Biol.* **204**, 231–245 (2014).

66. A. M. Roberts, F. M. Deacon, Gravitaxis in motile micro-organisms: The role of fore–Aft body asymmetry. *J. Fluid Mech.* **452**, 405–423 (2002).

67. S. H. Koenig, Brownian motion of an ellipsoid. A correction to Perrin's results. *Biopolymers* **14**, 2421–2423 (1975).

68. J. Happel, H. Brenner, *Low Reynolds Number Hydrodynamics*, vol. 1 of *Mechanics of Fluids and Transport Processes* (Springer Netherlands, 1981).

69. F. Perrin, Mouvement brownien d'un ellipsoide—I. Dispersion diélectrique pour des molécules ellipsoidales. *J. Phys. Radium.* **5**, 497–511 (1934).

70. M. Wada, A. Miyazaki, T. Fujii, On the mechanisms of diurnal vertical migration behavior of Heterosigma akashiwo (Raphidophyceae). *Plant Cell Physiol.* **26**, 431–436 (1985).

71. R. Milo, R. Phillips, *Cell Biology by the Numbers* (Garland Science, 2015).

72. M. Imran, M. Nadeem, Triacylglycerol composition, physico-chemical characteristics and oxidative stability of interesterified canola oil and fully hydrogenated cottonseed oil blends. *Lipids Health Dis.* **14**, 138 (2015).

73. S. L. Strom, E. L. Harvey, K. A. Fredrickson, S. Menden-Deuer, Broad salinity tolerance as a refuge from predation in the harmful raphidophyte alga Heterosigma akashiwo (Raphidophyceae). *J. Phycol.* **49**, 20–31 (2013).

74. E. Lauga, *The Fluid Dynamics of Cell Motility* (Cambridge Univ. Press, 2020).

75. I. R. Jenkinson, B. A. Biddanda, Bulk-phase viscoelastic properties of seawater relationship with plankton components. *J. Plankton Res.* **17**, 2251–2274 (1995).

76. I. R. Jenkinson, Bulk-phase viscoelastic properties of seawater. *Oceanol. Acta* **16**, 317–334 (1993).

77. G. Xu, K. S. Wilson, R. J. Okamoto, J.-Y. Shao, S. K. Dutcher, P. V. Bayly, Flexural rigidity and shear stiffness of flagella estimated from induced bends and counterbends. *Biophys. J.* **110**, 2759–2768 (2016).

78. J. Teran, L. Fauci, M. Shelley, Viscoelastic fluid response can increase the speed and efficiency of a free swimmer. *Phys. Rev. Lett.* **104**, 038101 (2010).
